# Supplementary material for: Qualitative exploration of women’s experiences of intramuscular pethidine or remifentanil patient-controlled analgesia for labour pain
Source: BMJ Open. 2019 Dec 23;9(12):e032203. doi: 10.1136/bmjopen-2019-032203 (PMC7008414; doi:10.1136/bmjopen-2019-032203)
Supplement: Author's manuscript [file bmjopen-2019-032203.draft_revisions.pdf]

BMJ Open is committed to open peer review. As part of this commitment we make the peer review history of every article we publish publicly available.

When an article is published we post the peer reviewers' comments and the authors' responses online. We also post the versions of the paper that were used during peer review. These are the versions that the peer review comments apply to.

The versions of the paper that follow are the versions that were submitted during the peer review process. They are not the versions of record or the final published versions. They should not be cited or distributed as the published version of this manuscript.

BMJ Open is an open access journal and the full, final, typeset and author-corrected version of record of the manuscript is available on our site with no access controls, subscription charges or pay-per-view fees (http://bmjopen.bmj.com).

If you have any questions on BMJ Open's open peer review process please email info.bmjopen@bmj.com

BMJ Open

A qualitative exploration of women's experiences of intramuscular pethidine or remifentanil patient controlled analgesia for labour pain

Journal:	*BMJ Open*
Manuscript ID	bmjopen-2019-032203
Article Type:	Research
Date Submitted by the Author:	07-Jun-2019

Complete List of Authors:	Moran, Victoria; UCLAN, ; University of Central Lancashire,<br>Thomson, Gillian; University of Central Lancashire, School of Health<br>Cook, Julie; University of Central Lancashire,<br>Storey, Hannah; University of Central Lancashire,<br>Beeson, Leanne; The University of Birmingham,<br>MacArthur, Christine; University of Birmingham, Public Health<br>Wilson, Matthew; University of Sheffield, Health Services Research

Keywords:	Maternal medicine < OBSTETRICS, labour, analgesia, pethidine, remifentanil, Pain management < ANAESTHETICS

SCHOLARONE™
Manuscripts

A qualitative exploration of women's experiences of intramuscular pethidine or remifentanil patient controlled analgesia for labour pain

Authors:

Hall Moran Victoria; Thomson Gillian, Cook Julie, Storey Hannah, Beeson Leanne, MacArthur Christine, Wilson Matthew

College of Health and Wellbeing, University of Central Lancashire, Preston, United Kingdom (Dr Victoria Hall Moran PhD, Dr Gillian Thomson PhD, Julie Cook BA, Hannah Storey MSc)

Institute of Applied Health Research, University of Birmingham, Birmingham, United Kingdom (Mrs Leanne Beeson BSc, Prof Christine MacArthur PhD)

School of Health and Related Research, University of Sheffield, Sheffield, United Kingdom (Dr Matthew J. A. Wilson MD)

Correspondence to: Dr Victoria Hall Moran
College of Health and Wellbeing, University of Central Lancashire, Preston PR1 2HE
Email: VLMoran@uclan.ac.uk
Telephone: 01772 89 3830

For peer review only

ABSTRACT:

Objectives

To explore women's birth experiences, of pain relief and infant feeding intentions and behaviours up to six weeks postpartum in women who received remifentanil or pethidine for labour pain.

Design

Qualitative postnatal sub-study to A Randomised Controlled Trial of Remifentanil intravenous patient Controlled analgesia (PCA) versus intramuscular pethidine for pain relief in labour (RESPITE).

Setting

Semi-structured audio-recorded telephone interviews conducted with women recruited to the RESPITE trial from seven UK hospitals at six weeks postpartum. Data were analysed using thematic analysis and overarching themes identified.

Participants

80 women consented and 49 (30 remifentanil group and 19 pethidine group) completed the interview.

Results

Eight themes were identified: 'birth expectations', 'negative physiological responses', 'effectiveness of pain relief', 'enabling a sense of control', 'cognitive effects', 'issues with drug administration', 'reflections for future choices' and 'impact on infant behaviour and breastfeeding'. Consistent with trial findings, many women who used remifentanil felt it provided effective pain relief, whereas women in the pethidine group expressed more mixed views. Side-effects were described in both groups with women using pethidine frequently reporting nausea. A number of women who used remifentanil complained of restricted movements due to technical aspects of drug administration, and fear of analgesia running out. Women described how remifentanil enabled them to maintain their ability to stay focussed during the birth. In both groups, women reported feeling disconnected from their labour. There appeared to be little difference in reported breastfeeding initiation and continuation between pethidine and remifentanil groups.

Conclusions

Qualitative insights from a follow-up study to atrial that explored experiences of intravenous remifentanil PCA with intra-muscular pethidine injection yielded little difference in breastfeeding experiences between groups. Remifentanil appeared to provide effective pain relief for many women whilst allowing them to remain alert and focussed during labour, although as with pethidine, some side-effects were noted.

Trial registration: The trial is registered at ISRCTN, number ISRCTN29654603.

For peer review only - http://bmjopen.bmj.com/site/about/guidelines.xhtml

For peer review only

Keywords: labour, childbirth, analgesia, pethidine, remifentanil, epidural, pain relief, postpartum, breastfeeding

Strengths and limitations of this study

- First study to explore in depth the experiences and infant feeding of women using intravenous remifentanil PCA for pain relief in labour
- Large number of interviews (49) conducted in multiple sites across England
- Fewer women recruited who had used pethidine (19) compared to remifentanil PCA (30)
- A high number of women also received Entonox® prior to randomisation and in combination with pethidine (41; 84%) and some progressed to epidural (11; 22%), which may have influenced their experiences.

INTRODUCTION

Women's experiences of and response to pain during labour are complex and may be influenced by many physiological and psychological factors, including fear, anxiety, prior experience and the degree of emotional support received in childbirth[1]. In line with the rise in the use of technology and intervention in childbirth, pharmacological pain management is widely offered to women in labour on maternity/obstetric units. Traditional pharmacological approaches for pain management include opioid analgesics, epidural analgesia and inhaled analgesia (50:50 oxygen and nitrous oxide). Epidural analgesia has been shown to be the most effective form of pain relief for labour, but may give rise to unwanted side effects. Systematic reviews of trials have identified that epidurals increase the duration of second stage labour and the risk of instrumental vaginal delivery[2].

Pethidine is the most commonly used opioid for labour pain relief in the UK[3]. Whilst pethidine is known to alleviate labour pain for some women, when compared to placebo, no clear differences have been observed in maternal satisfaction with pain relief or the number of women requesting an epidural[4]. Pethidine is known to cause maternal side-effects including nausea, vomiting and sedation[1], with potential for prolonged and more painful labour if the woman is unable to mobilise[3]. Opioids readily cross the placenta and neonatal respiratory depression and hypothermia remain concerns[1]. Intrapartum pethidine administration can have a detrimental effect on neonatal behaviour, reducing infant alertness, supressing the rooting and sucking reflex, and may shorten breastfeeding duration[5].

There is increasing interest in the role of remifentanil as an alternative opioid analgesia in labour[6]. Remifentanil is a potent, opioid medication with a short half-life, administered by patient-controlled analgesia (PCA), allowing the woman to have the drug as and when it is needed "On demand." A systematic review conducted in 2012, based on a few low quality small trials comparing remifentanil to pethidine administered by various routes, reported a reduction in progression to epidural with remifentanil[7]. A recent Cochrane review, again including only small poor quality trials, compared PCA remifentanil to alternative parenteral methods of pain relief in labour and found that remifentanil (PCA) provided more effective pain relief and women were more satisfied with pain relief compared to other opioids

For peer review only - http://bmjopen.bmj.com/site/about/guidelines.xhtml

administered intravenously, intramuscularly or using PCA[8]. Route of analgesia administration has been found to positively impact on women's experience of labour, with women who self-administered analgesia (intranasal fentanyl) reporting increased autonomy and satisfaction compared to women who relied on the midwife to administer analgesia, who were more often focused on the physical effect of the drug[9].

In this paper we report on a qualitative follow-up sub-study to an open-label randomised controlled trial undertaken in 14 UK maternity units. The main aim of the trial (RESPITE) was to compare intravenous remifentanil PCA with intra-muscular pethidine injection to determine whether remifentanil PCA reduced progression to epidural analgesia; also to consider whether it resulted in any adverse maternal or neonatal sequelae. The trial found that remifentanil intravenous PCA halved epidural conversion rate, compared to intramuscular pethidine[10]. The focus of this follow-up sub-study was to explore women's birth experiences, perceptions of their pain relief and infant feeding behaviours up to six weeks postpartum in women allocated to the pethidine, or remifentanil trial arms for labour pain.

METHODOLOGY

Design

Full details of the RESPITE trial are reported elsewhere[10]. This sub-study follow-up phase was an adjunct to the trial and comprised a qualitative exploratory study using semi-structured telephone interviews.

Ethics

Ethics/governance approval was obtained from a National Research Ethics Service Committee (NRES Committee East Midlands - Nottingham 2, reference number: 13/EM/0239), the lead authors' University (STEMH 304), and via the participating Trusts R&D departments. Participants gave written informed consent before taking part.

Recruitment

Fourteen UK maternity units took part in the RESPITE trial, and women in the qualitative sub-study were recruited from seven hospitals. Women who had participated in the trial were approached by a research midwife/nurse after childbirth, whilst an in-patient, and provided with a separate information leaflet about the follow-up phase. Women who were willing to take part in a telephone interview up to approximately 6 weeks post-partum were asked to sign a consent form. Participant contact details for consenting women were securely transferred to the research team, with all identifiable data stored on encrypted university files.

Verbal consent was reconfirmed at the time of interview. Women were recruited over a 16 month period and maternity units that recruited the most women within a one-month period received a £100 Love to Shop voucher.

Interviews

Semi-structured audio-recorded telephone interviews were conducted between 5-8 weeks post-partum by two of the authors (JC, HS). The interviews lasted between 20-30 minutes and explored women's pre-birth expectations for pain relief, their experiences and perceived impact of pain-relief on maternal or infant behaviours, and infant feeding. All women who took part in an interview received a £10 'Love to Shop' voucher to thank them for their participation.

For peer review only - http://bmjopen.bmj.com/site/about/guidelines.xhtml

Analysis

All interviews were transcribed verbatim, anonymised, and analysed with the help of NVIVO qualitative software. Thematic analysis of the entire data set was undertaken using Braun and Clark's approach[11]. Two authors (JC, HS) undertook this work through an iterative process of reading, identifying key codes, grouping codes into sub-themes and finally creating themes that were reflective of all views expressed. We paid particular attention to how women's experiences of pethidine and remifentanil, within the context of the trial, compared or differed. Analytical decisions were discussed and shared with all authors both periodically and at the end to agree final themes.

Patient and Public Involvement

Members of PRIME (Public and Researchers Involvement in Maternity and Early pregnancy group), a group of maternity service users convened by the University of Birmingham were involved in reviewing the participant information during study set up and were represented on the Trial Steering Committee for the RESPITE trial.

For peer review only

RESULTS

Eighty trial participants from seven hospitals agreed to take part in the RESPITE Postnatal sub-study. Despite numerous contact attempts, thirty-one women were not interviewed due to women not responding or declining to be interviewed. Forty-nine participants (30 remifentanil and 19 pethidine) took part in a telephone interview and were included for analysis.

Demographic information of participants is provided in Table 1. Eight women received pethidine before they entered the trial (trial eligibility stipulated no systemic opioid pain relief within the previous 4 hours), four of whom were subsequently assigned to the pethidine trial arm, and four to the remifentanil arm. The majority of women (41/49) received gas and air (Entonox®) pain relief in addition to the trial drug. Overall eleven women progressed to epidural: seven from the pethidine arm (conversion rate of 64%), and four from the remifentanil arm (conversion rate of 36%), somewhat higher than the conversion rates seen in the main trial (41% and 19% for pethidine and remifentanil respectively) (Wilson et al 2018). Of the eleven women who had an epidural, five women had a caesarean section; two from the pethidine arm and three from the remifentanil arm.

In the following section we report on eight themes relating to women's expectations and experiences of pain relief and the impact of analgesia on women's intentions and experiences of infant feeding across the cohort; with similarities and differences in the experiences of different forms of pain relief highlighted. A selection of participant quotes are included together with an identifier that reports that participant number and either an R (remifentanil) or P (pethidine) suffix to indicate their group allocation.

Birth Expectations

There were wide variations in women's approaches to birth planning. These ranged from some women having no clear plans; *'there wasn't any plans, no'* (14R) or how women *'just went with the flow'* (36R), to having very specific intentions; *'Yes I did a lot of planning, I knew*

For peer review only - http://bmjopen.bmj.com/site/about/guidelines.xhtml

exactly what I wanted' (50R). Often women's approaches to labour were related to their previous childbirth experience. Prior experiences of unfulfilled birth plans often led women to adopt a more flexible perspective due to the uncertainty of childbirth:

> Last time I was determined I wasn't going to have an epidural and then things didn't quite go according to plan so I had an epidural, so this time I decided to be, you know to go more with an open mind, because things can't be planned as much as you would like (36R)

The lack of a specific birth plan did not mean that women held no preferences or opinions regarding pain relief. Many women had actively researched pain relief options in a variety of ways, such as through antenatal classes, internet searches, and discussions with friends and family, although only two women had any prior knowledge of remifentanil. Some women considered pethidine to be the best available option:

> Well I'd looked at all the different types of pain relief beforehand and decided it was the one that I thought would be the best, so the pethidine. (15P)

However, others were less inclined to use pethidine due to the potential impact on the baby:

> It was really just the fact that it [pethidine] crossed over [to] the baby, and like I didn't really want him coming out sleepy. (55P)

Almost all the women interviewed were initially opposed to having an epidural due to reasons such as needle phobia, prior negative experience of epidural and vicarious reports of risk:

> *'I'd heard so many stories of people having nerve damage and complications'* (16P)

For a few of the women, their reticence reflected cultural expectations of how childbirth should be managed:

> I think it's a culture thing. Like, originally my parents are from [country], they're very against it [epidural]. I don't know if it's a myth or if there's any, or there is any research to back it up but they say it can cause lots of problems in the future and stuff like that. So we've kind of just been brought up around that. I've always kind of been told, oh yeah you know don't get that. (33P)

Negative physiological responses

Women from both trial arms reported a number of what they considered to be side effects and negative impacts of the analgesia on their physiological responses. While pethidine has a well-known sedative effect, for some of the women in our study, this was not related to effective pain relief, and rather became problematic when it rendered them incapable of staying awake in labour:

> Pethidine, but if I'm honest that did nothing apart from make me tired. [...] Well I just remember them giving me an injection and like I said it just made me, literally just trying not to fall asleep, that's how it felt. Like my eyes were really heavy and I were just nodding off but I was still in so much pain it just made things a bit worse if I'm honest because it didn't feel like it was numbing the pain at all it just literally made me want to go to sleep and I couldn't. [...] …because like I said it doesn't feel like it was

For peer review only - http://bmjopen.bmj.com/site/about/guidelines.xhtml

> any kind of pain relief it just made me drowsy like for me it was a bit pointless because I was still fully aware of what were going on with the pain it was just making it worse because I couldn't keep my eyes open. (73P – progressed to epidural)

Some women across both trial arms experienced drowsiness to the point that it interfered with their autonomy and mobility during labour:

> I was lying down and they told me not to move too much because obviously because it makes you that drowsy and that sleepy that it's best to stay in one position and just to press a button when you feel the pain. (45R)

Many of the women in the trial already had their mobility restricted by monitors, oxytocin infusion etc, and some of these women found that the delivery of remifentanil through a PCA device requiring continuous intravenous access further restricted their mobility:

> I found it [labour] very painful and I couldn't walk around, I couldn't really be on all fours very easily. All the things that I had imagined, I just couldn't do so because I was attached to the machine as well so yeah [...] No, I wasn't able to move around because I was attached to the, the baby was being monitored, and then when I had the cannula in, I think that restricted me as well. I wasn't sure exactly why I couldn't move around, whether it was the cannula or if it was because I was being monitored but yeah, I mean I didn't have long to go then once I had the remifentanil I don't think. Maybe an hour. (65R)

Several women in the pethidine arm reported experiencing vomiting or nausea, compared to only one woman in the remifentanil group. However, the woman reflected that it was difficult to disentangle the effects of remifentanil from the side effects of other analgesia:

> Perhaps a little bit drowsy because I know I was a bit in and out, and I was quite sick but I don't know if that was gas and air more than the actual drug. (74R)

Furthermore, for some women, their adverse physiological responses to remifentanil were only experienced when the analgesia *'ran out'*:

> The only thing I would say is because I was on it for quite a long time, the drip actually ran out and when the drip ran out, I had quite bad sickness and like body-shaking, my whole body was shaking, also it kind of, I think it got blocked at one point as well so that happened twice where like I was violently throwing up everywhere. (60R)

On some occasions, as reflected in the following quote, symptoms associated with remifentanil, led some women to cease using this form of pain relief:

> ...the first couple of times I did it okay, from the first go I didn't, I said I don't feel right and then after that I couldn't focus enough to even press the button when I needed to...It was just, I was just not with it, and then my hubby said oh can I press it for you, but the midwife said oh no it's got to be, it had to be me that did it. But I just couldn't, so I think in total I only pressed it probably four or five times and then decided to stop because I just didn't I just didn't like it at all. (36R)

For peer review only - http://bmjopen.bmj.com/site/about/guidelines.xhtml

Effectiveness of pain relief

There were mixed views regarding the effectiveness of pethidine for pain relief. Some women were happy with the pain relief obtained from pethidine; '*it was brilliant. I literally didn't feel a thing until after she was born* (38P)'. Women were satisfied with its effectiveness, particularly when used in combination with other pain-relief methods, e.g. gas and air:

> When I just had the gas and air it just felt like, wasn't really working at all, wasn't really doing anything but then obviously the two together just sort of took the edge off a little bit. Obviously I remember still feeling a bit of pain but it's sort of bearable if you like, a bit more bearable. Yeah, definitely felt the difference. (52P)

From a counter perspective, half of the women who received pethidine reported that their pain relief had been ineffective. These women complained about how they could *'feel everything'* and how it had made *'no difference'* to the intensity of their contractions:

> I didn't find the pethidine any help. It didn't help me at all [...] like, she said that it would like ease the contractions off, you know like the pain, but I didn't feel, I could still feel the pain when she, after the pethidine got injected in I could still feel everything [...] It might help other women but it didn't help me. (79P)

In contrast, only a few women found remifentanil ineffective as pain relief. However, this minority of accounts were generally related to delivery of the medication, or its inability to prevent pain caused by the baby's position in utero:

> As the labour progressed, because my son was back-to-back, a lot of the pain was in my back and I found it helped with the contractions but not necessarily the back pain in between the contractions. (58R)

Overall, there was a notable enthusiasm for remifentanil among participants. Some women described experiencing 'no pain' after its administration, whereas others referred to how it made the pain more bearable:

> Yes, it did. It definitely did help because before that it was just getting really unbearable. It was just so unbearable, I just couldn't, I felt as if I wanted to die [laughs], I know that's an exaggeration but that's how it feels at that point where you just can't bear that pain but when I took that medication or pain relief, it took that pain away. Obviously as soon as I felt that contraction coming, press the buzzer and it's like more than half of it were gone and it were just, it just slowly eases it all off and it were quite good. It was bearable then. (59R)

Enabling a sense of control

One of the key benefits of remifentanil highlighted by women was how it provided pain relief *and* maintained their ability to stay alert, awake or focussed during the birth:

> I still remember being very alert and having to listen to what the midwives were saying, you know push and everything so I was still very alert and able to do what I needed to do [...] Oh yeah, no I was still massively aware of what was going on and everything, so yeah. (44R)

For peer review only - http://bmjopen.bmj.com/site/about/guidelines.xhtml

For some women, being connected to a cannula did not substantially restrict their ability to move from the bed during labour, which, as reflected in the quote below, could have a positive impact on women's agency and self-efficacy:

> That's what I wanted to do anyway. I wanted a water birth. I wanted to be active. As soon as I got into labour anyway, before I had that medication, I didn't want to be on the bed, I hated being on the bed, being stuck with, restricted. I didn't like it, I just wanted to be able to move and it started giving me a little bit more confidence as well, you know, I just, not even, obviously I didn't care, I just wanted to get her out. I couldn't have done it without that [remifentanil], I really couldn't. (50R)

While women often needed time and practice to get into the rhythm of delivering remifentanil via the PCA, once this process was mastered it became a valued tool to retain a sense of control:

> I don't remember being in pain really and I just remember sort of, it was quite good because I was focussing on the light, thinking, right okay wait for the light, you know, it kind of gave me something to sort of focus on and think, right okay, push the button. And it definitely takes the pain away when you hit it. Yeah, so I think yeah it was good because you could self-administer it so you kind of knew that when, you were thinking right okay so I know now when I hit the button, the pain's going to go in a minute so like it could be a second or whatever, so yeah, I thought that was good. I did think it was a good drug. (56R)

Several women talked about how the PCA enabled them to use pain relief selectively as required. In this sense, they saw remifentanil as an opportunity to actively manage their own labour:

> But it was just so nice that you could be in control of it and it was one of those things that I didn't have to have it all the time. So, for example, with contractions, I'd press the button on the first, you know, with one contraction, and then I wouldn't have it for a couple of times because I'd be in control of my body, if that makes sense. [...] And for me, it was just what I wanted, I could feel, I was in control of me, I was in control of my body. I knew what was going on in my surroundings. (1R)

This capacity for self-regulation was supported by the immediate impact of remifentanil on women's physiological responses:

> But I noticed as well, when I pressed the buzzer, it worked straightaway, you know, the pain… [...] I just noticed like when I pressed the buzzer, you know, when I pressed the buzzer, you know, for pain relief, it was like instant, yes, the pain just like went. (12R)

The ability to maintain focus was strongly linked to the short-acting effect of remifentanil. The fact that each dose of remifentanil wore off quickly was experienced as empowering. One participant found that discontinuing use of the PCA quickly re-established the control she felt she was lacking:

> I really feel like when it came to the time of needing to push and things, when you're having to do sort of certain things at certain times, I needed to, I would have struggled if I had still been taking it because I couldn't kind of concentrate on anything, whereas

For peer review only - http://bmjopen.bmj.com/site/about/guidelines.xhtml

> because I'd stopped it and it was pretty much out of my system I was able to focus more. So I don't think it was in my system for very long. (36R)

On occasion, these positive effects were contrasted with women's previous experience of using pethidine:

> It was, obviously, because you've got control of it. So, you know, you were able to press it when you needed it. Whereas like, if you have the pethidine and that, once you've been given it you've got no control over how it's going to work. (14R)

While some of the women who had received pethidine made positive comments about its impact on their sense of control during labour, this was often in the context of providing respite to manage their labour pains:

> So I had the pain relief for a good few hours which had kind of given me a bit of respite actually because I was getting quite tired and quite, I really needed pain relief so when I got that then it just gave me a little bit of respite...(6R)

Cognitive effects

Some women, particularly in the remifentanil group, reported cognitive effects consistent with systemic opioids. For some, this was a negative experience, associated with a sense of vulnerability, where they felt unable to focus or process external stimuli and could, as reflected below, negate the potential salutary effects of medication use on labour pains:

> I was, I don't really know the technical term, almost knocked out by it, it made me really sort of, I don't know if that's the right word but I was really out of it, I couldn't focus, it was almost as, like, it was like it was too strong for me and they reduced the dose of it but I was still, I couldn't, you know the midwife was trying to ask me questions and give me instructions as they do, it's ongoing, and I really couldn't, I couldn't focus because I was almost sort of not in the room as it were, so for me that sort of, sort of negated any positive effect it would have had on the pain relief side of things. (36R)

Whereas while other women described feeling *'out of it'*, or *'away with the fairies'* after remifentanil administration, this was not always seen as being entirely negative:

> I would say it did take away my control and my sort of presence in the process so, you know at one point I could hear my mum and husband talking about me but couldn't voice an answer to them...Now that did mean that I was, you know, in a lovely place, and you know, I wasn't feeling any pain at that point but it meant that I was almost removed from the labour process. (58R)

A number of women in the remifentanil group also reflected on how their confused or altered state of mind was observed by their birth partners:

> I mean my husband said that I was talking about the car insurance, I was saying to him, 'Is the car insured?'...I was having a conversation about the car insurance and he was like, this is a weird time to talk about the car insurance (56R).

> I was in kind of in a completely different zone I think. My mum and husband were there and said that I'd gone from being in incredible pain to just really blissed out. (58R)

For peer review only - http://bmjopen.bmj.com/site/about/guidelines.xhtml

For both pain relief medications, women likened the effects of the analgesia to heavy alcohol consumption:

> To be honest I felt incredibly drunk or to the same effect. (55P)

> It was like being very, very, drunk. (76R)

A further negative impact reported by women with respect to both forms of pain relief, related to how the analgesia had impacted on their memories of labour and childbirth:

> I must admit within, literally within I've no idea because I was out of it. [...] So then, again I don't know how long I was on it for. […] Or maybe she was crying. I can't remember. I can't remember, sadly. (44R)

Issues with drug administration

On some occasions, difficulties were experienced due to the potential or actual unavailability of remifentanil. For example, some women described feeling *'stressed out'* due to concerns about whether the analgesia (drip bag) would run out:

> The pain relief it was brilliant, really, really good for about three hours and then the effects started to wear off. I don't know if the contractions were getting worse but I was clicking it a lot more than I had originally and I was running out of it and I was getting a bit stressed out because I was worried that I was going to run out. (32R)

In some instances, remifentanil was only reinstated after what women felt was a long delay (if at all), leaving them unexpectedly without pain relief:

> …but to leave someone without, there probably was a bit of pain relief in me but that ten minutes was a long ten minutes. Yeah, it's quite traumatising. (63R)

One woman reported that the issue of remifentanil running out had contributed to her using epidural:

> I ended up going on to have an epidural because my medication had finished out and what happened was my baby's heart rate kept dipping in and out. So they were a bit concerned and, obviously, I was still in pain after that, then I wasn't fully dilated. So I needed something to, to take the edge off, basically, so I did go in for an epidural, yes. (7R)

Several women also reported difficulties in opportunities to receive pain relief when the cannula became detached:

> I'd managed to rip my thingy out, the cannula thing so I didn't have it through the last bit basically because she said, 'oh I can't get it put back in, you'll just have to go on gas and air for the last bit'. (44R)

For some women the physiological effects of the remifentanil interfered with their ability to use the PCA to deliver the pain relief for each contraction:

For peer review only - http://bmjopen.bmj.com/site/about/guidelines.xhtml

> The other thing was, when I was using the button this is, it goes straight to your head, you know, and I was so woozy, when my next contraction would come, I would forget to press the button. And that contraction would feel absolutely dire because obviously the contraction before it had taken the edge off, and you know the midwife had to keep telling me [name] you need to press the button, and I was like, oh OK, OK. But I was in and out of it if I'm honest, but it was good, it was good. (48R)

Whereas for others, such as reflected in the quote below, the stability of the cannula directly affected their mobility:

> When I lifted up my hand it never went in properly, it had come up with an error message. So I had to make sure my hand was down when I pressed the button, which is a bit difficult when you're in contractions...No, I told them it wasn't working but they just told me to keep my hand down. (10R)

Reflections for future choices

Some of the women who received pethidine reported that they would opt for this form of pain relief in a future birth, even in occasions when the analgesia had not been wholly effective:

> It didn't get rid of the pain, but I would have it again...Yes, I wouldn't even think about it, I'd just have it next time...Obviously I'd go for the gas and air again if I could and then if it was like I was told I couldn't have it then I would go for the pethidine. (69P)

In converse, others were adamant that they would not use pethidine again, i.e. *'I hope I never have to have it* [pethidine] *again'* (55P) and would, where possible chose an alternative such as remifentanil:

> If hell froze over and I got pregnant again, I'd definitely have, I'd request remifentanil from the get go. (25P)

Similar mixed responses were also evident amongst women who received remifentanil. From one perspective, a number of women referred to how they would *'definitely'* use it again:

> I definitely felt I needed it so I would definitely have it again and I would definitely choose the same form of pain relief if it was offered to me again. I felt like it really worked for me. It definitely gave me what I needed and what I wanted from pain relief so I would definitely do the same again. (39R)

And would recommend it to others:

> I've also said to any other friends...if you are offered it [remifentanil], go for it because it's brilliant. (1R)

Whereas other women, notably all of whom had experienced adverse responses, would either request pethidine or make concerted efforts to not use pain relief:

> Gas and air yeah I'd have gas and air, again unless I needed it epidural no. I don't think any of the trial drugs or the drug I had before I wouldn't have. So just gas and

For peer review only - http://bmjopen.bmj.com/site/about/guidelines.xhtml

air...But me I don’t think I’d do it again. It didn’t agree with me, and yeah I don’t think I’d take it myself. (29R)

I think I would just stick to the pethidine this time because remifentanil, yeah it helped me but if it doesn’t stay in my body for the time I need it, because the remifentanil, just as when you press the button it comes into you whereas I need something that’s in me for longer to help me and unfortunately for me it only worked for a short time, so probably in the future I would probably just stick to pethidine. I wouldn’t go for epidural or anything. (34R)

Impact on infant behaviour and breastfeeding

The majority of women (33/49) expressed a prior intention to breastfeed their babies, whilst twelve women intended to formula feed and four had made no plans prior to their baby’s birth (see Table 1). Within the first hour of birth 47% (14/30) women in the remifentanil group and 42% (8/19) women in the pethidine group breastfed. By the time of interview (approximately 6 weeks postnatal), 47% (14/30) of women in the remifentanil group and 26% (5/19) in the pethidine group were either exclusively breastfeeding or mixed feeding their infants. A number of reasons were given for an earlier than expected cessation of breastfeeding, including level of support received, feeding difficulties such as infant tongue-tie and inverted nipples, and health problems for the mother (e.g. lupus, heart condition, mental health issues) or the baby (need for antibiotics, cleft palate, inadequate weight gain). None of the participants felt that the pain relief medication they received influenced their decision to cease breastfeeding.

Women in both groups, reported feeling exhausted immediately following the birth, and how this affected their initial interactions with their baby. However, as the effects of analgesia, and particularly remifentanil, dissipate after a short period of time, these complaints are likely to be attributed to the length and exertions associated with labour, rather than pain relief analgesia per se:

The first day, obviously, the night that he was born, I didn’t [have skin to skin], after he was born I was just asleep. When my mum come that evening, that afternoon, so she was there and my husband was there, and that day I was asleep. (11P)

Uncomfortable, tired, drowsy like even the nurses had to take over sometimes to feed my son in the night because I was that drowsy still and I couldn't, I just couldn't take any control it was a case of I was falling asleep with him on me and stuff like that because I was just so tired. (17R)

Other women felt so weak that they needed help from their birth partners to hold their babies:

So he was born, they were saying, give him milk, they put him on to my breast and I was giving him the milk but my arms didn’t have that much strength to hold him, my husband had to help me out. (11P)

One woman who had progressed to epidural reflected on her increasing frustration at not being able to breastfeed her baby:

I was so exhausted I couldn't really move because of my epidural and I was getting really tired and a bit emotional and everything so it wasn't, breastfeeding wasn't working basically (44R)

For peer review only - http://bmjopen.bmj.com/site/about/guidelines.xhtml

DISCUSSION

This study aimed to explore women's experiences of remifentanil and pethidine use for pain relief during labour and infant feeding behaviours up to approximately 6 weeks postpartum. The findings revealed that, whilst many women had actively researched pain relief options prior to their birth and had either formulated birth plans or had decided to adopt a more flexible approach to pain management, most were unfamiliar with the opioid remifentanil until they were informed about the trial. The majority of women who had used remifentanil felt that it had provided effective pain relief. This was not the case with women in the pethidine group, who had more mixed views, with many reporting that pethidine was completely ineffective at relieving their pain. This was consistent with the findings of the trial which showed that women's views of their pain relief effectiveness and average pain scores were significantly better in the remifentanil arm[10]. These insights also support a recent study by Fleet at colleagues[9] who compared women's use of pethidine and fentanyl (delivered on an intranasal or subcutaneous) basis. This study found that women who received fentanyl provided more positive, and less negative responses when compared to those who received pethidine[9]. However, it is important to note that while effective pain management has become an essential component of the care plan for expectant mothers, women's perceptions of pain are affected by physiological and/or psychological issues (e.g. fear, anxiety)[12], as well as the nature of the woman-provider relationship[13].

Women using either form of pain relief reported experiencing a number of side effects, although these tended to differ according to the analgesia used. Women using pethidine frequently reported feeling nauseated, consistent with the findings of Fleet et al[9], whereas this was only reported by one woman who had used remifentanil. Women in both groups reported that the opioid made them feel drowsy, although this seemed to be less pronounced in the remifentanil group. These insights support the trial findings[10] and the work of others such as Volikas and Male[14] in regard to the sedative effects of opioid based forms of pain relief.

A number of negative comments arose relating to the nature of remifentanil administration. Women in the remifentanil group described how the PCA restricted their movement, and for a small number, this resulted in pain (as a result of technically flawed intravenous cannulation) and frustration. Others experienced anxiety or difficulties due to the potential or actual unavailability of remifentanil. Women reported feeling anxious about whether the analgesia (drip bag) would run out and, on occasions when it did, they experienced significant delay in its reinstatement. On some occasions women considered that this may have contributed to their progression to epidural. Other qualitative research has also highlighted how opioids administered too late or wore off too early created fear and anxiety[9,15]. It is important to note that these technical difficulties are not inevitable components of PCA, and are amenable to resolution.

One of the key advantages of remifentanil highlighted by women was how it provided pain relief and maintained their ability to stay focussed during the birth. Several women referred to how PCA enabled them to use pain relief selectively as required. In this sense, they saw remifentanil as an opportunity to actively manage their own labour (not just the pain), helping them to find reference points for their own understanding of their progression and tolerance to

For peer review only - http://bmjopen.bmj.com/site/about/guidelines.xhtml

pain. The ability to maintain focus was probably related to the short-acting effect of remifentanil, which enhanced their sense of control. The association between the use of pharmacological pain relief and increased sense of control has also been reported by others[9,16,17].

A further notable finding, as reflected by others[9,18] related to women experiencing a cognitive impact of the analgesics. Some women reported feeling disconnected from their labour and unable to focus or process external stimuli. Some women felt that remifentanil impacted on their memory and affected their ability to recall their baby's birth. While this was not found in Fleet and colleagues' study[9], these differences may be related to additional factors such as levels of fear, and dosage of the medication, or the fact that more women in our study received remifentanil than pethidine.

In relation to infant feeding, it appeared that the majority of women intended to breastfeed their babies (67%), particularly those within the remifentanil group (73%) when compared with those in the pethidine group (60%). The trial findings showed no difference in breastfeeding within an hour of birth in the two trial arms[10]; with a similar pattern noted amongst those in this sub-study. Forty seven percent of women in the remifentanil and 42% of women in the pethidine arms initiating breastfeeding within the first hour after birth. At around 6 weeks postnatal, the rates of 'any' breastfeeding were 26% for women who used pethidine compared to 46% of women in the remifentanil group. While these variations may be attributable to the level of postnatal support for breastfeeding across the different areas, the findings do support those by Fleet and colleagues[9] in that women who used remifentanil had fewer breastfeeding difficulties.

This study is the first to explore the experiences of women using intravenous remifentanil PCA for pain relief in labour in depth, by formal, qualitative methodology. The study comprised a qualitative sub-study to a multi-centre RCT that compared intravenous remifentanil PCA with intra-muscular pethidine for pain relief in labour. The strengths of this study are that to date, there is minimal qualitative research to understand women's experiences of opioid use during pregnancy, and the large number of interviews conducted in multiple sites across England. The limitations relate to the relatively fewer women recruited who were randomised to pethidine and it may be that women who held stronger views towards the use of analgesia (positive or negative) took part in the study. The high attrition rates for the study highlight challenges intrinsic to early recruitment into postpartum studies, for example women may not have been fully cognisant of what they were agreeing to, and also difficulties in organising data collection when women are dealing with the demands of a new baby. It is also important to reflect that 41 (84%) of our sample received Entonox® prior to randomisation and in combination with pethidine and 11 (22%) progressed to epidural which may have influenced their experiences.

CONCLUSIONS

Qualitative insights from a follow-up sub-study to an open-label trial that compared intravenous remifentanil PCA with intra-muscular pethidine injection highlighted valuable differences. Overall, more women found remifentanil to be an effective pain relief, when compared with the mixed views of women who received pethidine. All women experienced side effects although these differed between groups, with a number of these difficulties associated with remifentanil related to drug administration rather than impact of the analgesia.

For peer review only - http://bmjopen.bmj.com/site/about/guidelines.xhtml

While negative experiences of remifentanil included restricted movements due to drug administration, a key advantage of this form of pain relief was how its short-lasting nature enabled women to remain focussed during the birth. Overall, while there was little difference in breastfeeding initiation rates, there was an indication that women who received remifentanil were more likely to continue breastfeeding. While these variations may be attributable to different numbers of women included from the trial groups and the level of postnatal support for breastfeeding across the different geographical areas, it may be that women who used remifentanil had fewer breastfeeding difficulties.

List of abbreviations

PCA (Patient Controlled Analgesia), RESPITE (A Randomised Controlled Trial of Remifentanil intravenous patient Controlled analgesia (PCA) versus intramuscular pethidine for pain relief in labour), ISRCTN (International Standard Randomised Controlled Trial Number), R&D (Research and Development)

DECLARATIONS

Funding statement

This work was supported by the Healthy Children Project, Inc. The RESPITE trial was funded by the National Institute for Health (NIHR) Research Clinician Scientist Award (NIHR ref: CS-11-030). CM is part funded by NIHR Collaborations for Leadership in Applied Health Research and Care West Midlands. The views expressed are those of the author(s) and not necessarily those of the NIHR or the Department of Health and Social Care.

Competing interests

The authors declare no competing interests.

Authors' contributions

The idea for the postnatal sub-study originated from VHM and GT and they secured funding to conduct it. VHM and GT performed the literature search and were responsible for the study design, with input from MW, CM and LB. Study conduct and data collection was led by VHM, GT, JC and HS with contributions from MW, CM and LB. Study analysis and was performed by JC and HS with supervision from VHM and GT. All authors were involved in data interpretation. Writing of the paper was led by VHM, GT, JC and HS with input from MW, CM and LB.

Acknowledgements

Professor Jane Daniels contributed to the trial design and conduct.

For peer review only - http://bmjopen.bmj.com/site/about/guidelines.xhtml

We thank the women who participated in the RESPITE post-natal sub-study. Finally, we express our gratitude to our National Health Service colleagues who supported recruitment for the study.

References

1 Jones L, Othman M, Dowswell T, Alfirevic Z, Gates S, Newburn M, Jordan S, Lavender T, Neilson JP. *Pain management for women in labour: an overview of systematic reviews. Cochrane Database of Systematic Reviews 2012, Issue 3. Art. No.: CD009234. DOI: 10.1002/14651858.CD009234.pub2.*
2 Anim-Somuah M, Smyth RMD, Cyna AM, Cuthbert A. Epidural versus non-epidural or no analgesia for pain management in labour. Cochrane Database of Systematic Reviews 2018, Issue 5. Art. No.: CD000331. DOI: 10.1002/14651858.CD000331.pub4.
3 Lawrence A, Lewis L, Hofmeyr GJ, Dowswell T, Styles C. Maternal positions and mobility during first stage labour. Cochrane Database of Systematic Reviews 2009*, Issue* 2. *DOI:* 10.1002/14651858.CD003934.pub2
4 Smith LA, Burns E, Cuthbert A. Parenteral opioids for maternal pain management in labour. Cochrane Database of Systematic Reviews 2018, Issue 6. Art. No.: CD007396. DOI: 10.1002/14651858.CD007396.pub3
5 Burchell, T., Coster, S. and Norman, I., 2016. The effect of intrapartum pethidine on breastfeeding: a scoping review. *Evidence Based Midwifery*, *14*, pp.49-56.

6 Kranke, P., Girard, T., Lavand'homme, P., Melber, A., Jokinen, J., Muellenbach, R.M., Wirbelauer, J. and Hönig, A., 2013. Must we press on until a young mother dies? Remifentanil patient controlled analgesia in labour may not be suited as a "poor man's epidural". *BMC pregnancy and childbirth*, *13*(1), p.139.
7 Schnabel A, Hahn N, Broscheit J, Muellenbach RM, Rieger L, Roewer N, et al. Remifentanil for labour analgesia: a meta-analysis of randomised controlled trials. European Journal of Anaesthesiology (EJA). 2012;29(4):177-85.
8 Weibel, S., Jelting, Y., Afshari, A., Pace, N.L., Eberhart, L.H., Jokinen, J., Artmann, T. and Kranke, P., 2017. Patient-controlled analgesia with remifentanil versus alternative parenteral methods for pain management in labour. *Cochrane Database of Systematic Reviews*, (4).
9 Fleet J, Jones M, Belin I: Taking the alternative route: Women's experience of intranasal fentanyl, subcutaneous fentanyl or intramuscular pethidine for labour analgesia. Midwifery 2017, 53:15-19.
10 Wilson, M.J., MacArthur, C., Hewitt, C.A., Handley, K., Gao, F., Beeson, L., Daniels, J. and Group, R.T.C., 2018. Intravenous remifentanil patient-controlled analgesia versus intramuscular pethidine for pain relief in labour (RESPITE): an open-label, multicentre, randomised controlled trial. *The Lancet*, *392*(10148), pp.662-672
11 Braun, V. and Clarke, V., 2006. Using thematic analysis in psychology. *Qualitative research in psychology*, *3*(2), pp.77-101.
12 Lundgren I, Dahlberg K: Women's experience of pain during childbirth. Midwifery 1998, 14(2):105-110.
13 Karlsdottir S, Halldorsdottir S, Lundgren I: The third paradigm in labour pain preparation and management: the childbearing woman's paradigm. Scandinavian Journal of Caring Sciences 2014, 28(2):315-327.
14 Volikas I, Male D. A comparison of pethidine and remifentanil patient-controlled analgesia in labour. Int J Obstet Anesth. 2001;10(2):86-90.

For peer review only - http://bmjopen.bmj.com/site/about/guidelines.xhtml

15 Nikkola E, Läärä A, Hinkka S, Ekblad U, Kero P, Salonen M: Patient-controlled epidural analgesia in labor does not always improve maternal satisfaction. Acta Obstet Gynecol Scand 2006, 85(2):188-194
16 Doering K, Patterson J, Griffiths C: Japanese women's experiences of pharmacological pain relief in New Zealand. Women and Birth 2014, 27(2):121-125
17 Lally, J.E., Thomson, R.G., MacPhail, S. and Exley, C., 2014. Pain relief in labour: a qualitative study to determine how to support women to make decisions about pain relief in labour. *BMC pregnancy and childbirth*, *14*(1), p.6.
18 Jantjes L, Strumpher J, Kotze W: The experience of childbirth in first-time mothers who received narcotic analgesics during the first stage of labour. Curationis 2007, 30(2):82-90.

For peer review only

For peer review only - http://bmjopen.bmj.com/site/about/guidelines.xhtml

	Remifentanil (n=30)	Pethidine (N=19)	Total (n=49)
**Age of women (years)**			
Mean (SD)	29.3 (5.2)	25.9 (4.5)	28.0 (5.2)
Range	19-39	17-34	17-39
**Ethnicity**			
White	23	14	37
Asian (Indian)	2	1	3
Asian (Pakistani)	2	4	6
Mixed	1	0	1
Other	2	0	2
**Marital status**			
Married/living with partner	30	17	47
Single	0	2	2
**Parity**			
Nulliparous	21	13	34
Multiparous	9	6	15
**Type of birth**			
Vaginal	26	15	41
Caesarean	4	4	8
**Additional pain relief administered:**			
Epidural	4	7	11
Pethidine	4	4	8
Entonox	24	17	41
**Infant feeding intention:**			
Breastfeeding	21	12	33
Formula feeding	6	6	12
No pre-birth plans	3	1	4

For peer review only

Infant feeding initiation within the first hour post-birth:			
Breastfeeding	14	8	22
Formula feeding	16	11	27
**Infant feeding status at ~6 weeks**			
Exclusive breastfeeding	7	0	7
Formula feeding	16	14	30
Mixed feeding	7	5	12

Table 1 Demographic information of study participants

For peer review only

Reporting checklist for qualitative study.

Based on the SRQR guidelines.

Instructions to authors

Complete this checklist by entering the page numbers from your manuscript where readers will find each of the items listed below.

Your article may not currently address all the items on the checklist. Please modify your text to include the missing information. If you are certain that an item does not apply, please write "n/a" and provide a short explanation.

Upload your completed checklist as an extra file when you submit to a journal.

In your methods section, say that you used the SRQRreporting guidelines, and cite them as:

O'Brien BC, Harris IB, Beckman TJ, Reed DA, Cook DA. Standards for reporting qualitative research: a synthesis of recommendations. Acad Med. 2014;89(9):1245-1251.

		Reporting Item	Page Number
**Title**			
	#1	Concise description of the nature and topic of the study identifying the study as qualitative or indicating the approach (e.g. ethnography, grounded theory) or data collection methods (e.g. interview, focus group) is recommended	1

For peer review only

Abstract

	#2	Summary of the key elements of the study using the abstract format of the intended publication; typically includes background, purpose, methods, results and conclusions	2

Introduction

Problem formulation	#3	Description and signifcance of the problem / phenomenon studied: review of relevant theory and empirical work; problem statement	3-4
Purpose or research question	#4	Purpose of the study and specific objectives or questions	4

Methods

Qualitative approach and research paradigm	#5	Qualitative approach (e.g. ethnography, grounded theory, case study, phenomenolgy, narrative research) and guiding theory if appropriate; identifying the research paradigm (e.g. postpositivist, constructivist / interpretivist) is also recommended; rationale. The rationale should briefly discuss the justification for choosing that theory, approach, method or technique rather than other options available; the assumptions and limitations implicit in those choices and how those choices influence study conclusions and transferability.	4

For peer review only

		As appropriate the rationale for several items might be discussed together.	
Researcher characteristics and reflexivity	#6	Researchers' characteristics that may influence the research, including personal attributes, qualifications / experience, relationship with participants, assumptions and / or presuppositions; potential or actual interaction between researchers' characteristics and the research questions, approach, methods, results and / or transferability	4
Context	#7	Setting / site and salient contextual factors; rationale	4
Sampling strategy	#8	How and why research participants, documents, or events were selected; criteria for deciding when no further sampling was necessary (e.g. sampling saturation); rationale	4
Ethical issues pertaining to human subjects	#9	Documentation of approval by an appropriate ethics review board and participant consent, or explanation for lack thereof; other confidentiality and data security issues	4
Data collection methods	#10	Types of data collected; details of data collection procedures including (as appropriate) start and stop dates of data collection and analysis, iterative process, triangulation of sources / methods, and modification of procedures in response to evolving study findings; rationale	4

For peer review only

Data collection instruments and technologies	#11	Description of instruments (e.g. interview guides, questionnaires) and devices (e.g. audio recorders) used for data collection; if / how the instruments(s) changed over the course of the study	4
Units of study	#12	Number and relevant characteristics of participants, documents, or events included in the study; level of participation (could be reported in results)	5
Data processing	#13	Methods for processing data prior to and during analysis, including transcription, data entry, data management and security, verification of data integrity, data coding, and anonymisation / deidentification of excerpts	4
Data analysis	#14	Process by which inferences, themes, etc. were identified and developed, including the researchers involved in data analysis; usually references a specific paradigm or approach; rationale	5
Techniques to enhance trustworthiness	#15	Techniques to enhance trustworthiness and credibility of data analysis (e.g. member checking, audit trail, triangulation); rationale	5

Results/findings

Syntheses and interpretation	#16	Main findings (e.g. interpretations, inferences, and themes); might include development of a theory or model, or integration with prior research or theory	5-14

For peer review only

Links to empirical data	#17	Evidence (e.g. quotes, field notes, text excerpts, photographs) to substantiate analytic findings	5-14
**Discussion**			
Intergration with prior work, implications, transferability and contribution(s) to the field	#18	Short summary of main findings; explanation of how findings and conclusions connect to, support, elaborate on, or challenge conclusions of earlier scholarship; discussion of scope of application / generalizability; identification of unique contributions(s) to scholarship in a discipline or field	14
Limitations	#19	Trustworthiness and limitations of findings	15
**Other**			
Conflicts of interest	#20	Potential sources of influence of perceived influence on study conduct and conclusions; how these were managed	16
Funding	#21	Sources of funding and other support; role of funders in data collection, interpretation and reporting	16

The SRQR checklist is distributed with permission of Wolters Kluwer © 2014 by the Association of American Medical Colleges. This checklist was completed on 07. June 2019 using https://www.goodreports.org/, a tool made by the EQUATOR Network in collaboration with Penelope.ai

For peer review only

BMJ Open

A qualitative exploration of women's experiences of intramuscular pethidine or remifentanil patient controlled analgesia for labour pain

Journal:	*BMJ Open*
Manuscript ID	bmjopen-2019-032203.R1
Article Type:	Original research
Date Submitted by the Author:	14-Oct-2019
Complete List of Authors:	Moran, Victoria; UCLAN, ; University of Central Lancashire,<br>Thomson, Gillian; University of Central Lancashire, School of Health<br>Cook, Julie; University of Central Lancashire,<br>Storey, Hannah; University of Central Lancashire,<br>Beeson, Leanne; The University of Birmingham,<br>MacArthur, Christine; University of Birmingham, Public Health<br>Wilson, Matthew; University of Sheffield, Health Services Research
**Primary Subject Heading**:	Obstetrics and gynaecology
Secondary Subject Heading:	Qualitative research
Keywords:	Maternal medicine < OBSTETRICS, labour, analgesia, pethidine, remifentanil, Pain management < ANAESTHETICS

SCHOLARONE™
Manuscripts

A qualitative exploration of women's experiences of intramuscular pethidine or remifentanil patient controlled analgesia for labour pain

Authors:

Hall Moran Victoria; Thomson Gillian, Cook Julie, Storey Hannah, Beeson Leanne, MacArthur Christine, Wilson Matthew

Hall Moran Victoria College of Health and Wellbeing, University of Central Lancashire, Preston, United Kingdom

Thomson Gillian, College of Health and Wellbeing, University of Central Lancashire, Preston, United Kingdom

Cook Julie, College of Health and Wellbeing, University of Central Lancashire, Preston, United Kingdom

Storey Hannah, College of Health and Wellbeing, University of Central Lancashire, Preston, United Kingdom

Beeson Leanne, Institute of Applied Health Research, University of Birmingham, Birmingham, United Kingdom

MacArthur Christine, Institute of Applied Health Research, University of Birmingham, Birmingham, United Kingdom

Wilson Matthew, School of Health and Related Research, University of Sheffield, Sheffield, United Kingdom

Correspondence to: Dr Victoria Hall Moran
College of Health and Wellbeing, University of Central Lancashire, Preston PR1 2HE
Email: VLMoran@uclan.ac.uk
Telephone: 01772 89 3830

WORD COUNT: 7539

For peer review only

ABSTRACT:

Objectives

To explore women's birth experiences, of pain relief and infant feeding behaviours up to six weeks postpartum in women who received remifentanil or pethidine for labour pain.

Design

Qualitative postnatal sub-study to the randomised controlled trial of remifentanil Intravenous patient controlled analgesia (PCA) versus intramuscular pethidine for pain relief in labour (RESPITE). Semi-structured interviews were conducted at six weeks postpartum. Data were analysed using thematic analysis and overarching themes identified

Setting

Telephone interviews conducted with women recruited to the RESPITE trial from seven UK hospitals.

Participants

80 women consented and 49 (30 remifentanil group and 19 pethidine group) completed the interview.

Results

Eight themes were identified, which encompassed women's plans for pain management in the antenatal period (*Birth Expectations*) through to her preferences for pain relief in future births (*Reflections for Future Choices*)., , , , ‘’. Consistent with trial findings, many women who used remifentanil felt it provided effective pain relief (*Effectiveness of Pain Relief*), whereas women in the pethidine group expressed more mixed views. Side-effects were described in both groups with women using pethidine frequently reporting nausea (*Negative Physiological Responses*) and women using remifentanil describing more cognitive effects (*Cognitive Effects*). A number of women who used remifentanil complained of restricted movements due to technical aspects of drug administration, and fear of analgesia running out (*Issues with Drug Administration*). Women described how remifentanil enabled them to maintain their ability to stay focussed during the birth (*Enabling a Sense of Control*). In both groups, women reported feeling disconnected from their labour. There appeared to be little difference in reported breastfeeding

For peer review only - http://bmjopen.bmj.com/site/about/guidelines.xhtml

initiation and continuation between pethidine and remifentanil groups (*Impact on Infant Behaviour and Breastfeeding*).

Conclusions

Qualitative insights from a follow-up study to a trial which explored experiences of intravenous remifentanil PCA with intra-muscular pethidine injection yielded little difference in breastfeeding initiation rates between groups, although there was an indication that women who received remifentanil may be more likely to continue breastfeeding. Remifentanil appeared to provide effective pain relief for many women whilst allowing them to remain alert and focussed during labour, although as with pethidine, some side-effects were noted.

For peer review only

Trial registration: The RESPITE trial is registered at ISRCTN, number ISRCTN29654603.

Keywords: labour, childbirth, analgesia, pethidine, remifentanil, epidural analgesia, pain relief, postpartum, breastfeeding

Contributor statement:

Strengths and limitations of this study

- To our knowledge, this is the first study to explore in depth the experiences and infant feeding behaviours of women using intravenous remifentanil PCA for pain relief in labour
- Substantial number of women (49) interviewed from multiple sites across England
- Fewer women recruited who had used pethidine (19) compared to remifentanil PCA (30)
- A high number of women also received Entonox® prior to randomisation and in combination with pethidine (41; 84%) and some progressed to epidural analgesia (11; 22%), which may have influenced their experiences.

INTRODUCTION

Women's experiences of and response to pain during labour are complex, affected by multiple physiological and psychosocial factors, and consequently many women in labour require some form of pain relief [1]. In line with the rise in the use of technology and intervention in childbirth, pharmacological pain management is widely offered to women in labour on maternity/obstetric units. Traditional pharmacological approaches for pain management include opioid analgesics, epidural analgesia and inhaled analgesia (50:50 oxygen and nitrous oxide). Epidural analgesia has been shown to be the most effective form of pain relief for labour, but may give rise to unwanted side effects. Systematic reviews of trials have identified that epidural analgesics increase the duration of second stage labour and the risk of instrumental vaginal delivery[2].

For peer review only - http://bmjopen.bmj.com/site/about/guidelines.xhtml

It has been reported that up to 25% of women in the UK use pethidine or a similar opioid during labour [3] Whilst pethidine is known to alleviate labour pain for some women, when compared to placebo, no clear differences have been observed in maternal satisfaction with pain relief or the number of women requesting an epidural analgesic[4]. Pethidine is known to cause maternal side-effects including nausea, vomiting and sedation[1]. Opioids readily cross the placenta and neonatal respiratory depression and hypothermia remain concerns[1]. Intrapartum pethidine administration may have a detrimental effect on neonatal behaviour, reducing infant alertness, supressing the rooting and sucking reflex, and may shorten breastfeeding duration[5].

There is increasing interest in the role of remifentanil as an alternative opioid analgesia in labour[6]. Remifentanil is a potent opioid medication with a short half-life, administered by patient-controlled analgesia (PCA), allowing the woman to have the drug as and when it is needed on demand. A systematic review conducted in 2012, based on a few low quality small trials comparing remifentanil to pethidine administered by various routes, reported a reduction in progression to epidural analgesia with remifentanil[7]. A recent Cochrane review, again including only small poor quality trials, compared PCA remifentanil to alternative parenteral methods of pain relief in labour and found that remifentanil (PCA) provided more effective pain relief and women were more satisfied with pain relief compared to other opioids administered intravenously, intramuscularly or using PCA[8]. Known side effects of remifentanil include respiratory depression, nausea, pruritus, and decreased heart rate and blood pressure, although there are currently too few studies of remifentanil PCA use in labour to draw definite conclusion with respect to side effects for women and newborns[8.] Route of analgesia administration has been found to positively impact on women's experience of labour, with women who self-administered analgesia (intranasal fentanyl) reporting increased autonomy and satisfaction compared to women who relied on the midwife to administer analgesia, who were more often focused on the physical effect of the drug[9]. The influence of remifentanil on infant feeding behaviours remains largely unexplored.

In this paper we report on a qualitative follow-up sub-study to an open-label randomised controlled trial undertaken in 14 UK maternity units. The main aim of the trial (RESPITE) was to compare intravenous remifentanil PCA with intra-muscular pethidine injection to determine whether remifentanil PCA reduced progression to epidural analgesia; also to consider whether it resulted in any adverse maternal or neonatal sequelae. The trial found that remifentanil intravenous PCA halved epidural analgesia conversion rate, compared to intramuscular pethidine[10]. The importance of combining qualitative evidence that explores the views and experiences of service users alongside quantitative evidence to inform safety, effectiveness and cost of interventions to inform antenatal and intrapartum guidelines has been recognised by the World Health Organisation [11,12] . The focus of this follow-up sub-study was to explore women's birth experiences, perceptions of their pain relief and infant feeding behaviours up to six weeks postpartum in women allocated to the pethidine, or remifentanil trial arms for labour pain.

METHODOLOGY

Design

Full details of the RESPITE trial are reported elsewhere[10]. This sub-study follow-up phase was an adjunct to the trial and comprised a qualitative exploratory study using semi-structured telephone interviews.

For peer review only - http://bmjopen.bmj.com/site/about/guidelines.xhtml

Ethics
Ethics/governance approval was obtained from a National Research Ethics Service Committee (NRES Committee East Midlands - Nottingham 2, reference number: 13/EM/0239), the lead authors' University (STEMH 304), and via the participating Trusts R&D departments. Participants gave written informed consent before taking part.

Recruitment
Fourteen UK obstetric led maternity units took part in the RESPITE trial where all women booked for delivery at participating centres were informed about the study at their antenatal visits. Women were eligible for the trial if they had met the following inclusion criteria: 16 years or older and beyond 37 weeks' gestation, with a singleton live baby, in cephalic presentation, who were in established labour (defined as regular painful contractions irrespective of cervical dilatation), intending vaginal birth, and were not participating in any other drug trial. For the qualitative sub-study, women who participated in the trial were approached by a research midwife/nurse after childbirth, whilst an in-patient, and provided with a separate information leaflet. Women who were willing to take part in a telephone interview up to approximately 6 weeks post-partum were asked to sign a consent form. Participant contact details for consenting women were securely transferred to the research team, with all identifiable data stored on encrypted university files.

Verbal consent was reconfirmed at the time of interview. Women were recruited over a 16 month period (between June 2015 and September 2016) and maternity units that recruited the most women within a one-month period received a £100 Love to Shop voucher.

Interviews
Semi-structured audio-recorded telephone interviews were conducted between 5-8 weeks post-partum by two research assistants (JC, HS). Telephone interviews provided participants with the opportunity to disclose intimate and sensitive experiences without feeling uncomfortable. The interviews lasted between 20-30 minutes and explored women's pre-birth expectations for pain relief, their experiences and perceived impact of pain-relief on maternal or infant behaviours, and infant feeding. Questions such as, "how did the pain relief affect your labour? (e.g. emotionally, physically)?", "how did you feel after the birth?", and "how was your baby following the birth?", were posed. All women who took part in an interview received a £10 'Love to Shop' voucher to thank them for their participation.

Analysis
All interviews were transcribed verbatim, anonymised, and uploaded into NVIVO qualitative software[13]. Data collection and analysis was undertaken concurrently, and Braun and Clark's thematic approach[14] was undertaken to identify patterned meaning across the dataset. Two authors (JC, HS) undertook this work (under the guidance of VHM and GT) through an iterative process of reading, identifying key codes, grouping codes into sub-themes and finally creating themes that were reflective of all views expressed. We paid particular attention to how women's experiences of pethidine and remifentanil, within the context of the trial, compared or differed. Analytical decisions were discussed and shared with all authors both periodically and at the end to agree final themes.

Patient and Public Involvement

For peer review only - http://bmjopen.bmj.com/site/about/guidelines.xhtml

Members of PRIME (Public and Researchers Involvement in Maternity and Early pregnancy group), a group of maternity service users convened by the University of Birmingham were involved in reviewing the participant information during study set up and were represented on the Trial Steering Committee for the RESPITE trial.

RESULTS

Eighty trial participants from seven hospitals agreed to take part in the RESPITE Postnatal sub-study. Whilst all fourteen sites participating in the RESPITE trial were eligible for the sub-study, only seven hospitals successfully consented participants who progressed to interview. Four maternity units received £100 in shopping vouchers as reward for recruiting the most women within a one-month period. Despite numerous contact attempts, 23 women were not interviewed due to women being unavailable during the timeframe of the study or due to language barriers. Eight women asked to be withdrawn from the study prior to interview. Forty-nine participants (30 remifentanil and 19 pethidine) took part in a telephone interview and were included for analysis.

Demographic information of participants is provided in Table 1. Eight women received pethidine before they entered the trial (trial eligibility stipulated no systemic opioid pain relief within the previous 4 hours), four of whom were subsequently assigned to the pethidine trial arm, and four to the remifentanil arm. The majority of women (41/49) received gas and air (Entonox®) pain relief in addition to the trial drug. Overall eleven women progressed to epidural analgesia: seven from the pethidine arm (conversion rate of 64%), and four from the remifentanil arm (conversion rate of 36%), somewhat higher than the conversion rates seen in the main trial (41% and 19% for pethidine and remifentanil respectively)[10]. Of the eleven women who had an epidural analgesic, five women had a caesarean section; two from the pethidine arm and three from the remifentanil arm. A further three women had a caesarean section without having had epidural analgesia.

Table 1 Demographic information of study participants

	Remifentanil (n=30)	Pethidine (N=19)	Total (n=49)
**Age of women (years)**			
Mean (SD)	29.3 (5.2)	25.9 (4.5)	28.0 (5.2)
Range	19-39	17-34	17-39
**Ethnicity**			
White	23	14	37
Asian (Indian)	2	1	3
Asian (Pakistani)	2	4	6
Mixed	1	0	1
Other	2	0	2
**Marital status**<br>Married/living with partner	30<br>0	17<br>2	47<br>2

For peer review only - http://bmjopen.bmj.com/site/about/guidelines.xhtml

For peer review only

Single			
**Parity**<br><br>Nulliparous<br>Parous	 <br>21<br>9	 <br>13<br>6	 <br>34<br>15
**Type of birth**<br>Vaginal<br>Caesarean	 26<br>4	 15<br>4	 41<br>8
**Additional pain relief administered:**<br>Epidural analgesia<br>Pethidine<br>Entonox	 <br>4<br>4<br>24	 <br>7<br>4<br>17	 <br>11<br>8<br>41
**Infant feeding intention:**<br>Breastfeeding<br>Formula feeding<br>No pre-birth plans	 21<br>6<br>3	 12<br>6<br>1	 33<br>12<br>4
Infant feeding initiation within the first hour post-birth:<br>Breastfeeding<br>Formula feeding	 <br><br>14<br>16	 <br><br>8<br>11	 <br><br>22<br>27
**Infant feeding status at ~6 weeks**<br>Exclusive breastfeeding<br>Formula feeding<br>Mixed feeding	 <br>7<br>16<br>7	 <br>0<br>14<br>5	 <br>7<br>30<br>12

In the following section we report on eight inter-linking themes relating to women's expectations and experiences of pain relief and the impact of analgesia on women's experiences of infant feeding across the cohort; with similarities and differences in the experiences of different forms of pain relief highlighted. As no stark differences were found between the responses of nulliparous and parous women no differentiation was made in the findings. A selection of participant quotes are included together with an identifier that reports that participant number and either an R (remifentanil) or P (pethidine) suffix to indicate their group allocation.

The eight themes identified encompassed women's plans for pain management in the antenatal period (*Birth Expectations*) through to how her experience of using either remifentanil or pethidine may influence her future preferences for pain relief in labour (*Reflections for Future Choices*). Four themes centred around women's physiological and cognitive response to the analgesics (*Negative Physiological Response*; *Cognitive Effects*), and reflections on how effective pethidine and remifentanil were for pain relief (*Effectiveness of Pain Relief*) or pain management (*Enabling a Sense of Control*). Additional themes highlighted common issues with the administration of the analgesics which may have impacted negatively on her experience (*Issues with Drug Administration*) and women's reflections on whether they felt the

For peer review only - http://bmjopen.bmj.com/site/about/guidelines.xhtml

For peer review only

analgesics had an influence on their newborn and the establishment of breastfeeding (*Impact on Infant Behaviour and Breastfeeding*).

Birth Expectations

There were wide variations in women's approaches to birth planning. These ranged from some women having no clear plans; *'there wasn't any plans, no*' (14R) or how women '*just went with the flow'* (36R), to having very specific intentions; '*Yes I did a lot of planning, I knew exactly what I wanted*' (50R). Often women's approaches to labour were related to their previous childbirth experience. Prior experiences of unfulfilled birth plans often led women to adopt a more flexible perspective due to the uncertainty of childbirth:

> Last time I was determined I wasn't going to have an epidural and then things didn't quite go according to plan so I had an epidural, so this time I decided to be, you know to go more with an open mind, because things can't be planned as much as you would like (36R)

The lack of a specific birth plan did not mean that women held no preferences or opinions regarding pain relief. Many women had actively researched pain relief options in a variety of ways, such as through antenatal classes, internet searches, and discussions with friends and family, although only two women had any prior knowledge of remifentanil. Some women considered pethidine to be the best available option:

> Well I'd looked at all the different types of pain relief beforehand and decided it was the one that I thought would be the best, so the pethidine. (15P)

However, others were less inclined to use pethidine due to the potential impact on the baby:

> It was really just the fact that it [pethidine] crossed over [to] the baby, and like I didn't really want him coming out sleepy. (55P)

Almost all the women interviewed were initially opposed to having an epidural analgesic due to reasons such as needle phobia, prior negative experience of epidural analgesia and vicarious reports of risk:

> I'd heard so many stories of people having nerve damage and complications (16P)

For a few of the women, their reticence reflected cultural expectations of how childbirth should be managed:

> I think it's a culture thing. Like, originally my parents are from [country], they're very against it [epidural]. I don't know if it's a myth or if there's any, or there is any research to back it up but they say it can cause lots of problems in the future and stuff like that. So we've kind of just been brought up around that. I've always kind of been told, oh yeah you know don't get that. (33P)

Negative physiological responses

Women from both trial arms reported what they considered to be side effects and negative impacts of the analgesia on their physiological responses. While pethidine has a well-known

For peer review only - http://bmjopen.bmj.com/site/about/guidelines.xhtml

sedative effect, for some of the women in our study, this was not related to effective pain relief, and rather became problematic when it rendered them incapable of staying awake in labour:

> Well I just remember them giving me an injection and […]it just made me, literally just trying not to fall asleep, that's how it felt. Like my eyes were really heavy and I were just nodding off but I was still in so much pain it just made things a bit worse if I'm honest because it didn't feel like it was numbing the pain at all it just literally made me want to go to sleep and I couldn't. [...] … (73P – progressed to epidural analgesia)

Some women across both trial arms experienced drowsiness to the point that it interfered with their autonomy and mobility during labour:

> I was lying down and they told me not to move too much because obviously because it makes you that drowsy and that sleepy that it's best to stay in one position and just to press a button when you feel the pain. (45R)

Many of the women in the trial already had their mobility restricted by monitors, oxytocin infusion etc, and some of these women found that the delivery of remifentanil through a PCA device requiring continuous intravenous access further restricted their mobility:

> I found it [labour] very painful and I couldn't walk around, I couldn't really be on all fours very easily. All the things that I had imagined, I just couldn't do so because I was attached to the machine as well so yeah [...] (65R)

Several women in the pethidine arm reported experiencing vomiting or nausea, compared to only one woman in the remifentanil group. However, the woman reflected that it was difficult to disentangle the effects of remifentanil from the side effects of other analgesia:

> Perhaps a little bit drowsy because I know I was a bit in and out, and I was quite sick but I don't know if that was gas and air more than the actual drug. (74R)

Furthermore, for some women, their adverse physiological responses to remifentanil were only experienced when the analgesia *'ran out'*:

> The only thing I would say is because I was on it for quite a long time, the drip actually ran out and when the drip ran out, I had quite bad sickness and like body-shaking, my whole body was shaking, also it kind of, I think it got blocked at one point as well so that happened twice where like I was violently throwing up everywhere. (60R)

On some occasions, as reflected in the following quote, symptoms associated with remifentanil, led some women to cease using this form of pain relief:

> ...the first couple of times I did it okay, from the first go I didn't, I said I don't feel right and then after that I couldn't focus enough to even press the button when I needed to... I was just not with it, and then my hubby said oh can I press it for you, but the midwife said oh no it's got to be, it had to be me that did it. But I just couldn't, so I think in total I only pressed it probably four or five times and then decided to stop because I just didn't I just didn't like it at all. (36R)

For peer review only - http://bmjopen.bmj.com/site/about/guidelines.xhtml

Effectiveness of pain relief

There were mixed views regarding the effectiveness of pethidine for pain relief. Some women were happy with the pain relief obtained from pethidine; '*it was brilliant. I literally didn't feel a thing until after she was born* (38P)'. Women were satisfied with its effectiveness, particularly when used in combination with other pain-relief methods, e.g. gas and air:

> When I just had the gas and air it just felt like, wasn't really working at all, wasn't really doing anything but then obviously the two together just sort of took the edge off a little bit. Obviously I remember still feeling a bit of pain but it's sort of bearable if you like, a bit more bearable. Yeah, definitely felt the difference. (52P)

From a counter perspective, half of the women who received pethidine reported that their pain relief had been ineffective. These women complained about how they could *'feel everything'* and how it had made *'no difference'* to the intensity of their contractions:

> I didn't find the pethidine any help. It didn't help me at all [...] like, she said that it would like ease the contractions off, you know like the pain, but I didn't feel, I could still feel the pain when she, after the pethidine got injected in I could still feel everything [...] It might help other women but it didn't help me. (79P)

In contrast, only a few women found remifentanil ineffective as pain relief. However, this minority of accounts were generally related to delivery of the medication, or its inability to prevent pain caused by the baby's position in utero:

> As the labour progressed, because my son was back-to-back, a lot of the pain was in my back and I found it helped with the contractions but not necessarily the back pain in between the contractions. (58R)

Overall, there was a notable enthusiasm for remifentanil among participants. Some women described experiencing 'no pain' after its administration, whereas others referred to how it made the pain more bearable:

> Yes, it did. It definitely did help because before that it was just getting really unbearable. It was just so unbearable, I just couldn't, I felt as if I wanted to die [laughs], I know that's an exaggeration but that's how it feels at that point where you just can't bear that pain but when I took that medication or pain relief, it took that pain away. Obviously as soon as I felt that contraction coming, press the buzzer and it's like more than half of it were gone and it were just, it just slowly eases it all off and it were quite good. It was bearable then. (59R)

Enabling a sense of control

One of the key benefits of remifentanil highlighted by women was how it provided pain relief *and* maintained their ability to stay alert, awake or focussed during the birth:

> I still remember being very alert and having to listen to what the midwives were saying, you know push and everything so I was still very alert and able to do what I needed to do [...](44R)

For peer review only - http://bmjopen.bmj.com/site/about/guidelines.xhtml

For some women, being connected to a cannula did not substantially restrict their ability to move from the bed during labour, which, as reflected in the quote below, could have a positive impact on women's agency and self-efficacy:

> That's what I wanted to do anyway. I wanted a water birth. I wanted to be active. As soon as I got into labour anyway, before I had that medication, I didn't want to be on the bed, I hated being on the bed, being stuck with, restricted. I didn't like it, I just wanted to be able to move and it started giving me a little bit more confidence as well, you know, I just, not even, obviously I didn't care, I just wanted to get her out. I couldn't have done it without that [remifentanil], I really couldn't. (50R)

Women often needed time and practice to get into the rhythm of delivering remifentanil via the PCA. Once mastered several women talked about how the PCA enabled them to retain a sense of control by actively managing their own labour:

> But it was just so nice that you could be in control of it and it was one of those things that I didn't have to have it all the time. So, for example, with contractions, I'd press the button on the first, you know, with one contraction, and then I wouldn't have it for a couple of times because I'd be in control of my body, if that makes sense. [...] And for me, it was just what I wanted, I could feel, I was in control of me, I was in control of my body. I knew what was going on in my surroundings. (1R)

This capacity for self-regulation was supported by the immediate impact of remifentanil on women's physiological responses:

> But I noticed as well, when I pressed the buzzer, it worked straightaway, you know, the pain… [...] I just noticed like when I pressed the buzzer, you know, when I pressed the buzzer, you know, for pain relief, it was like instant, yes, the pain just like went. (12R)

The ability to maintain focus was strongly linked to the short-acting effect of remifentanil. The fact that each dose of remifentanil wore off quickly was experienced as empowering. One participant found that discontinuing use of the PCA quickly re-established the control she felt she was lacking:

> I really feel like when it came to the time of needing to push and things, when you're having to do sort of certain things at certain times, I needed to, I would have struggled if I had still been taking it because I couldn't kind of concentrate on anything, whereas because I'd stopped it and it was pretty much out of my system I was able to focus more. So I don't think it was in my system for very long. (36R)

On occasion, these positive effects were contrasted with women's previous experience of using pethidine:

> It was, obviously, because you've got control of it. So, you know, you were able to press it when you needed it. Whereas like, if you have the pethidine and that, once you've been given it you've got no control over how it's going to work. (14R)

For peer review only - http://bmjopen.bmj.com/site/about/guidelines.xhtml

While some of the women who had received pethidine made positive comments about its impact on their sense of control during labour, this was often in the context of providing respite to manage their labour pains:

> So I had the pain relief for a good few hours which had kind of given me a bit of respite actually because I was getting quite tired and quite, I really needed pain relief so when I got that then it just gave me a little bit of respite...(6R)

Cognitive effects

Some women, particularly in the remifentanil group, reported cognitive effects consistent with systemic opioids. For some, this was a negative experience, associated with a sense of vulnerability, where they felt unable to focus or process external stimuli and could, as reflected below, negate the potential salutary effects of medication use on labour pains:

> I was, I don't really know the technical term, almost knocked out by it, it made me really sort of, I don't know if that's the right word but I was really out of it, I couldn't focus, it was almost […]like it was too strong for me and they reduced the dose of it but […]the midwife was trying to ask me questions and give me instructions as they do, it's ongoing, and I really couldn't[…] focus because I was almost sort of not in the room as it were, so for me that sort of, sort of negated any positive effect it would have had on the pain relief side of things. (36R)

Whereas while other women described feeling *'out of it'*, or *'away with the fairies'* after remifentanil administration, this was not always seen as being entirely negative:

> I would say it did take away my control and my sort of presence in the process so, you know at one point I could hear my mum and husband talking about me but couldn't voice an answer to them...Now that did mean that I was, you know, in a lovely place, and I wasn't feeling any pain at that point but it meant that I was almost removed from the labour process. (58R)

A number of women in the remifentanil group also reflected on how their confused or altered state of mind was observed by their birth partners:

> I mean my husband said that I was talking about the car insurance, I was saying to him, 'Is the car insured?'...I was having a conversation about the car insurance and he was like, this is a weird time to talk about the car insurance (56R).

> I was in kind of in a completely different zone I think. My mum and husband were there and said that I'd gone from being in incredible pain to just really blissed out. (58R)

For both pain relief medications, women likened the effects of the analgesia to heavy alcohol consumption:

> To be honest I felt incredibly drunk or to the same effect. (55P)

> It was like being very, very, drunk. (76R)

A further negative impact reported by women with respect to both forms of pain relief, related to how the analgesia had impacted on their memories of labour and childbirth:

For peer review only - http://bmjopen.bmj.com/site/about/guidelines.xhtml

> I must admit within, literally within I've no idea because I was out of it. [...] So then, again I don't know how long I was on it for. […] Or maybe she was crying. I can't remember. I can't remember, sadly. (44R)

Issues with drug administration

On some occasions, difficulties were experienced due to the potential or actual unavailability of remifentanil. For example, some women described feeling *'stressed out'* due to concerns about whether the analgesia (drip bag) would run out:

> The pain relief it was brilliant, really, really good for about three hours and then the effects started to wear off. I don't know if the contractions were getting worse but I was clicking it a lot more than I had originally and I was running out of it and I was getting a bit stressed out because I was worried that I was going to run out. (32R)

In some instances, remifentanil was only reinstated after what women felt was a long delay (if at all), leaving them unexpectedly without pain relief:

> …but to leave someone without, there probably was a bit of pain relief in me but that ten minutes was a long ten minutes. Yeah, it's quite traumatising. (63R)

One woman reported that the issue of remifentanil running out had contributed to her using epidural analgesia:

> I ended up going on to have an epidural because my medication had finished out and what happened was my baby's heart rate kept dipping in and out. So they were a bit concerned and, obviously, I was still in pain after that, then I wasn't fully dilated. So I needed something to, to take the edge off, basically, so I did go in for an epidural, yes. (7R)

Several women also reported difficulties in opportunities to receive pain relief when the cannula became detached:

> I'd managed to rip my thingy out, the cannula thing so I didn't have it through the last bit basically because she said, 'oh I can't get it put back in, you'll just have to go on gas and air for the last bit'. (44R)

For some women the physiological effects of the remifentanil interfered with their ability to use the PCA to deliver the pain relief for each contraction:

> The other thing was, when I was using the button this is, it goes straight to your head, you know, and I was so woozy, when my next contraction would come, I would forget to press the button. And that contraction would feel absolutely dire because obviously the contraction before it had taken the edge off, and you know the midwife had to keep telling me [name] you need to press the button, and I was like, oh OK, OK. But I was in and out of it if I'm honest, but it was good, it was good. (48R)

Whereas for others, such as reflected in the quote below, the stability of the cannula directly affected their mobility:

For peer review only - http://bmjopen.bmj.com/site/about/guidelines.xhtml

> When I lifted up my hand it never went in properly, it had come up with an error message. So I had to make sure my hand was down when I pressed the button, which is a bit difficult when you're in contractions...No, I told them it wasn't working but they just told me to keep my hand down. (10R)

Reflections for future choices
Some of the women who received pethidine reported that they would opt for this form of pain relief in a future birth, even in occasions when the analgesia had not been wholly effective:

> It didn't get rid of the pain, but I would have it again...Yes, I wouldn't even think about it, I'd just have it next time...Obviously I'd go for the gas and air again if I could and then if it was like I was told I couldn't have it then I would go for the pethidine. (69P)

In converse, others were adamant that they would not use pethidine again, i.e. *'I hope I never have to have it* [pethidine] *again'* (55P) and would, where possible chose an alternative such as remifentanil:

> If hell froze over and I got pregnant again, I'd definitely have, I'd request remifentanil from the get go. (25P)

Similar mixed responses were also evident amongst women who received remifentanil. From one perspective, a number of women referred to how they would *'definitely'* use it again:

> I definitely felt I needed it so I would definitely have it again and I would definitely choose the same form of pain relief if it was offered to me again. I felt like it really worked for me. It definitely gave me what I needed and what I wanted from pain relief so I would definitely do the same again. (39R)

And would recommend it to others:

> I've also said to any other friends...if you are offered it [remifentanil], go for it because it's brilliant. (1R)

Whereas other women, notably all of whom had experienced adverse responses, would either request pethidine or make concerted efforts to not use pain relief:

> Gas and air yeah I'd have gas and air, again unless I needed it epidural no. I don't think any of the trial drugs or the drug I had before I wouldn't have. So just gas and air...But me I don't think I'd do it again. It didn't agree with me, and yeah I don't think I'd take it myself. (29R)

Impact on infant behaviour and breastfeeding
The majority of women (33/49) expressed a prior intention to breastfeed their babies, whilst twelve women intended to formula feed and four had made no plans prior to their baby's birth (see Table 1). Within the first hour of birth 47% (14/30) women in the remifentanil group and 42% (8/19) women in the pethidine group breastfed. By the time of interview (approximately 6 weeks postnatal), 47% (14/30) of women in the remifentanil group and 26% (5/19) in the pethidine group were either exclusively breastfeeding or mixed feeding their infants. A number

For peer review only - http://bmjopen.bmj.com/site/about/guidelines.xhtml

For peer review only

of reasons were given for an earlier than expected cessation of breastfeeding, including level of support received, feeding difficulties such as infant tongue-tie and inverted nipples, and health problems for the mother (e.g. lupus, heart condition, mental health issues) or the baby (need for antibiotics, cleft palate, inadequate weight gain). None of the participants felt that the pain relief medication they received influenced their decision to cease breastfeeding.

Women in both groups, reported feeling exhausted immediately following the birth, and how this affected their initial interactions with their baby. However, as the effects of analgesia, and particularly remifentanil, dissipate after a short period of time, these complaints are likely to be attributed to the length and exertions associated with labour, rather than pain relief analgesia per se:

> The first day, obviously, the night that he was born, I didn't [have skin to skin], after he was born I was just asleep. When my mum come that evening, that afternoon, so she was there and my husband was there, and that day I was asleep. (11P)

> Uncomfortable, tired, drowsy like even the nurses had to take over sometimes to feed my son in the night because I was that drowsy still and I couldn't, I just couldn't take any control it was a case of I was falling asleep with him on me and stuff like that because I was just so tired. (17R)

Other women felt so weak that they needed help from their birth partners to hold their babies:

> So he was born, they were saying, give him milk, they put him on to my breast and I was giving him the milk but my arms didn't have that much strength to hold him, my husband had to help me out. (11P)

One woman who had progressed to epidural analgesia reflected on her increasing frustration at not being able to breastfeed her baby:

> I was so exhausted I couldn't really move because of my epidural and I was getting really tired and a bit emotional and everything so it wasn't, breastfeeding wasn't working basically (44R)

DISCUSSION

This study aimed to explore women's experiences of remifentanil and pethidine use for pain relief during labour and infant feeding behaviours up to approximately 6 weeks postpartum. The findings revealed that, whilst many women had actively researched pain relief options prior to their birth and had either formulated birth plans or had decided to adopt a more flexible approach to pain management, most were unfamiliar with the opioid remifentanil until they were informed about the trial. The majority of women who had used remifentanil felt that it had provided effective pain relief. This was not the case with women in the pethidine group, who had more mixed views, with many reporting that pethidine was completely ineffective at relieving their pain. This was consistent with the findings of the trial which showed that women's views of their pain relief effectiveness and average pain scores were significantly better in the remifentanil arm[10]. These insights also support a recent study by Fleet and

For peer review only - http://bmjopen.bmj.com/site/about/guidelines.xhtml

colleagues[9] who compared women's use of pethidine and fentanyl (delivered on an intranasal or subcutaneous) basis. This study found that women who received fentanyl provided more positive, and less negative responses when compared to those who received pethidine[9]. However, it is important to note that while effective pain management has become an essential component of the care plan for expectant mothers, women's perceptions of pain are affected by physiological and/or psychological issues (e.g. fear, anxiety)[15], as well as the nature of the woman-provider relationship[16].

Women using either form of pain relief reported experiencing a number of side effects, although these tended to differ according to the analgesia used. Women using pethidine frequently reported feeling nauseated, consistent with the findings of Fleet and colleagues[9], whereas this was only reported by one woman who had used remifentanil. Women in both groups reported that the opioid made them feel drowsy, although this seemed to be less pronounced in the remifentanil group. These insights support the trial findings[10] and the work of others such as Volikas and Male[17] in regard to the sedative effects of opioid based forms of pain relief.

A number of negative comments arose relating to the nature of remifentanil administration. Women in the remifentanil group described how the PCA restricted their movement, and for a small number, this resulted in pain (as a result of technically flawed intravenous cannulation) and frustration. Others experienced anxiety or difficulties due to the potential or actual unavailability of remifentanil. Women reported feeling anxious about whether the analgesia (drip bag) would run out and, on occasions when it did, they experienced significant delay in its reinstatement. On some occasions women considered that this may have contributed to their progression to epidural analgesia. Other qualitative research has also highlighted how opioids administered too late or wore off too early created fear and anxiety[9,18]. It is important to note that these technical difficulties are not inevitable components of PCA, and are amenable to resolution.

One of the key advantages of remifentanil highlighted by women was how it provided pain relief and maintained their ability to stay focussed during the birth. Several women referred to how PCA enabled them to use pain relief selectively as required. In this sense, they saw remifentanil as an opportunity to stay in control and actively manage their own labour (not just the pain), helping them to find reference points for their own understanding of their progression and tolerance to pain. The ability to maintain focus was probably related to the short-acting effect of remifentanil, which enhanced their sense of control. The association between the use of pharmacological pain relief and increased sense of control has also been reported by others[9,12,19,20].

A further notable finding, as reflected by others[9,21] related to women experiencing a cognitive impact of the analgesics. Some women reported feeling disconnected from their labour and unable to focus or process external stimuli. Similar findings were also reported in a study by Whitburn and colleagues to explore women's experiences of labour pain. They found that an ability to retain focus during labour was associated with self-judgement and negative perspectives of pain [22]. Some women felt that remifentanil impacted on their memory and affected their ability to recall their baby's birth. While this was not found in Fleet and colleagues' study[9], these differences may be related to additional factors such as levels of fear, and dosage of the medication, or the fact that more women in our study received remifentanil than pethidine.

For peer review only - http://bmjopen.bmj.com/site/about/guidelines.xhtml

In relation to infant feeding, The main trial found no difference in breastfeeding within an hour of birth in the two trial arms[10]; with a similar pattern noted amongst those in this sub-study. Forty seven percent of women in the remifentanil and 42% of women in the pethidine arms initiating breastfeeding within the first hour after birth. At around 6 weeks postnatal, the rates of 'any' breastfeeding were 26% for women who used pethidine compared to 46% of women in the remifentanil group. While these variations may be attributable to the level of postnatal support for breastfeeding across the different areas, the findings do support those by Fleet and colleagues[23] in that women who used remifentanil had fewer breastfeeding difficulties. These differences may relate to the differential effects of analgesia on infant behaviours which may impact on establishment of sustained breastfeeding[24]. However, as there are many biopsychosocial factors that can influence the mother's infant feeding decisions, further research is needed.

For peer review only

To our knowledge, this is the first study to explore the experiences of women using intravenous remifentanil PCA for pain relief in labour in depth, by formal, qualitative methodology. The study comprised a qualitative sub-study to a multi-centre RCT that compared intravenous remifentanil PCA with intra-muscular pethidine for pain relief in labour. The strengths of this study are that to date, there is minimal qualitative research to understand women's experiences of opioid use during labour, and the substantial number of interviews conducted in multiple sites across England. The limitations relate to the relatively fewer women recruited who were randomised to pethidine and it may be that women who held stronger views towards the use of analgesia (positive or negative) took part in the study. The high attrition rates for the study highlight challenges intrinsic to early recruitment into postpartum studies, for example women may not have been fully cognisant of what they were agreeing to, and also difficulties in organising data collection when women are dealing with the demands of a new baby. It is also important to reflect that 41 (84%) of our sample received Entonox® prior to randomisation and in combination with pethidine and 11 (22%) progressed to epidural analgesia which may have influenced their experiences.

CONCLUSIONS

Qualitative insights from a follow-up sub-study to an open-label trial that compared intravenous remifentanil PCA with intra-muscular pethidine injection highlighted valuable differences. Overall, more women found remifentanil to be an effective pain relief, when compared with the mixed views of women who received pethidine. All women experienced side effects although these differed between groups, with a number of these difficulties associated with remifentanil related to drug administration rather than impact of the analgesia. While negative experiences of remifentanil included restricted movements due to drug administration, a key advantage of this form of pain relief was how its short-lasting nature enabled women to remain focussed during the birth. Overall, while there was little difference in breastfeeding initiation rates, there was an indication that women who received remifentanil were more likely to continue breastfeeding. While these variations may be attributable to different numbers of women included from the trial groups and the level of postnatal support for breastfeeding across the different geographical areas, it may be that women who used remifentanil had fewer breastfeeding difficulties.

For peer review only - http://bmjopen.bmj.com/site/about/guidelines.xhtml

List of abbreviations

PCA (Patient Controlled Analgesia), RESPITE (A Randomised Controlled Trial of Remifentanil intravenous patient Controlled analgesia (PCA) versus intramuscular pethidine for pain relief in labour), ISRCTN (International Standard Randomised Controlled Trial Number), R&D (Research and Development)

DECLARATIONS

Funding statement

This work was supported by the Healthy Children Project, Inc. The RESPITE trial was funded by the National Institute for Health (NIHR) Research Clinician Scientist Award (NIHR ref: CS-11-030). CM is part funded by NIHR Collaborations for Leadership in Applied Health Research and Care West Midlands. The views expressed are those of the author(s) and not necessarily those of the NIHR or the Department of Health and Social Care.

Data sharing statement

Data will not be made publicly available. For more information, please contact the corresponding author.

Competing interests

The authors declare no competing interests.

Contributor satement

The idea for the postnatal sub-study originated from VHM and GT and they secured funding to conduct it. VHM and GT performed the literature search and were responsible for the study design, with input from MW, CM and LB. Study conduct and data collection was led by VHM, GT, JC and HS with contributions from MW, CM and LB. Study analysis and was performed by JC and HS with supervision from VHM and GT. All authors were involved in data interpretation. Writing of the paper was led by VHM, GT, JC and HS with input from MW, CM and LB.

Acknowledgements

Professor Jane Daniels contributed to the trial design and conduct.

We thank the women who participated in the RESPITE post-natal sub-study. Finally, we express our gratitude to our National Health Service colleagues who supported recruitment for the study.

References

For peer review only

1 Jones L, Othman M, Dowswell T, Alfirevic Z, Gates S, Newburn M, Jordan S, Lavender T, Neilson JP. *Pain management for women in labour: an overview of systematic reviews. Cochrane Database of Systematic Reviews 2012, Issue 3. Art. No.: CD009234. DOI: 10.1002/14651858.CD009234.pub2.*
2 Anim-Somuah M, Smyth RMD, Cyna AM, Cuthbert A. Epidural versus non-epidural or no analgesia for pain management in labour. Cochrane Database of Systematic Reviews 2018, Issue 5. Art. No.: CD000331. DOI: 10.1002/14651858.CD000331.pub4.

3 Redshaw M, Henderson J. Safely Delivered: a National Survey of Women's Experience of Maternity Care 2014. Oxford: The National Perinatal Epidemiology Unit, University of Oxford, 2015.
4 Smith LA, Burns E, Cuthbert A. Parenteral opioids for maternal pain management in labour. Cochrane Database of Systematic Reviews 2018, Issue 6. Art. No.: CD007396. DOI: 10.1002/14651858.CD007396.pub3
5 Burchell, T., Coster, S. and Norman, I., 2016. The effect of intrapartum pethidine on breastfeeding: a scoping review. *Evidence Based Midwifery*, *14*, pp.49-56.
6 Kranke, P., Girard, T., Lavand'homme, P., Melber, A., Jokinen, J., Muellenbach, R.M., Wirbelauer, J. and Hönig, A., 2013. Must we press on until a young mother dies? Remifentanil patient controlled analgesia in labour may not be suited as a "poor man's epidural". *BMC pregnancy and childbirth*, *13*(1), p.139.
7 Schnabel A, Hahn N, Broscheit J, Muellenbach RM, Rieger L, Roewer N, et al. Remifentanil for labour analgesia: a meta-analysis of randomised controlled trials. European Journal of Anaesthesiology (EJA). 2012;29(4):177-85.
8 Weibel, S., Jelting, Y., Afshari, A., Pace, N.L., Eberhart, L.H., Jokinen, J., Artmann, T. and Kranke, P., 2017. Patient-controlled analgesia with remifentanil versus alternative parenteral methods for pain management in labour. *Cochrane Database of Systematic Reviews*, (4).
9 Fleet J, Jones M, Belin I: Taking the alternative route: Women's experience of intranasal fentanyl, subcutaneous fentanyl or intramuscular pethidine for labour analgesia. Midwifery 2017, 53:15-19.
10 Wilson, M.J., MacArthur, C., Hewitt, C.A., Handley, K., Gao, F., Beeson, L., Daniels, J. and Group, R.T.C., 2018. Intravenous remifentanil patient-controlled analgesia versus intramuscular pethidine for pain relief in labour (RESPITE): an open-label, multicentre, randomised controlled trial. *The Lancet*, *392*(10148), pp.662-672
11 Downe, S., Finlayson, K., Tunçalp, Ö. and Metin Gülmezoglu, A., 2016. What matters to women: a systematic scoping review to identify the processes and outcomes of antenatal care provision that are important to healthy pregnant women. *BJOG: An International Journal of Obstetrics & Gynaecology*, *123*(4), pp.529-539.

12 Thomson, G., Feeley, C., Moran, V.H., Downe, S. and Oladapo, O.T., 2019. Women's experiences of pharmacological and non-pharmacological pain relief methods for labour and childbirth: a qualitative systematic review. *Reproductive health*, *16*(1), p.71

13 NVIVO Software https://www.qsrinternational.com/nvivo

14 Braun, V. and Clarke, V., 2006. Using thematic analysis in psychology. *Qualitative research in psychology*, *3*(2), pp.77-101.
15 Lundgren I, Dahlberg K: Women's experience of pain during childbirth. Midwifery 1998, 14(2):105-110.
16 Karlsdottir S, Halldorsdottir S, Lundgren I: The third paradigm in labour pain preparation and management: the childbearing woman's paradigm. Scandinavian Journal of Caring Sciences 2014, 28(2):315-327.

For peer review only

17 Volikas I, Male D. A comparison of pethidine and remifentanil patient-controlled analgesia in labour. Int J Obstet Anesth. 2001;10(2):86-90.
18 Nikkola E, Läärä A, Hinkka S, Ekblad U, Kero P, Salonen M: Patient-controlled epidural analgesia in labor does not always improve maternal satisfaction. Acta Obstet Gynecol Scand 2006, 85(2):188-194
19 Doering K, Patterson J, Griffiths C: Japanese women's experiences of pharmacological pain relief in New Zealand. Women and Birth 2014, 27(2):121-125
20 Lally, J.E., Thomson, R.G., MacPhail, S. and Exley, C., 2014. Pain relief in labour: a qualitative study to determine how to support women to make decisions about pain relief in labour. *BMC pregnancy and childbirth*, *14*(1), p.6.
21 Jantjes L, Strumpher J, Kotze W: The experience of childbirth in first-time mothers who received narcotic analgesics during the first stage of labour. Curationis 2007, 30(2):82-90.
22 Whitburn LY, Jones LE, Davey M-A, Small R. Women's experiences of labour pain and the role of the mind: an exploratory study. Midwifery. 2014;30(9):1029-35
23 Fleet, J., Jones, M., Belan, I. The influence of intrapartum opioid use on breastfeeding experience at 6 weeks post partum: a secondary analysis. Midwifery 2017
50, 106–109
24 Ransjö-Arvidson, A.B., Matthiesen, A.S., Lilja, G., Nissen, E., Widström, A.M. and Uvnäs-Moberg, K., 2001. Maternal analgesia during labor disturbs newborn behavior: effects on breastfeeding, temperature, and crying. *Birth*, *28*(1), pp.5-12.

For peer review only

For peer review only - http://bmjopen.bmj.com/site/about/guidelines.xhtml

Reporting checklist for qualitative study.

Based on the SRQR guidelines.

Instructions to authors

Complete this checklist by entering the page numbers from your manuscript where readers will find each of the items listed below.

Your article may not currently address all the items on the checklist. Please modify your text to include the missing information. If you are certain that an item does not apply, please write "n/a" and provide a short explanation.

Upload your completed checklist as an extra file when you submit to a journal.

In your methods section, say that you used the SRQRreporting guidelines, and cite them as:

O'Brien BC, Harris IB, Beckman TJ, Reed DA, Cook DA. Standards for reporting qualitative research: a synthesis of recommendations. Acad Med. 2014;89(9):1245-1251.

		Reporting Item	Page Number
**Title**			
	#1	Concise description of the nature and topic of the study identifying the study as qualitative or indicating the approach (e.g. ethnography, grounded theory) or data collection methods (e.g. interview, focus group) is recommended	1

For peer review only

Abstract

	#2	Summary of the key elements of the study using the abstract format of the intended publication; typically includes background, purpose, methods, results and conclusions	2

Introduction

Problem formulation	#3	Description and signifcance of the problem / phenomenon studied: review of relevant theory and empirical work; problem statement	3-4
Purpose or research question	#4	Purpose of the study and specific objectives or questions	4

Methods

Qualitative approach and research paradigm	#5	Qualitative approach (e.g. ethnography, grounded theory, case study, phenomenolgy, narrative research) and guiding theory if appropriate; identifying the research paradigm (e.g. postpositivist, constructivist / interpretivist) is also recommended; rationale. The rationale should briefly discuss the justification for choosing that theory, approach, method or technique rather than other options available; the assumptions and limitations implicit in those choices and how those choices influence study conclusions and transferability.	4

For peer review only

		As appropriate the rationale for several items might be discussed together.	
Researcher characteristics and reflexivity	#6	Researchers' characteristics that may influence the research, including personal attributes, qualifications / experience, relationship with participants, assumptions and / or presuppositions; potential or actual interaction between researchers' characteristics and the research questions, approach, methods, results and / or transferability	4
Context	#7	Setting / site and salient contextual factors; rationale	4
Sampling strategy	#8	How and why research participants, documents, or events were selected; criteria for deciding when no further sampling was necessary (e.g. sampling saturation); rationale	4
Ethical issues pertaining to human subjects	#9	Documentation of approval by an appropriate ethics review board and participant consent, or explanation for lack thereof; other confidentiality and data security issues	4
Data collection methods	#10	Types of data collected; details of data collection procedures including (as appropriate) start and stop dates of data collection and analysis, iterative process, triangulation of sources / methods, and modification of procedures in response to evolving study findings; rationale	4

For peer review only

Data collection instruments and technologies	#11	Description of instruments (e.g. interview guides, questionnaires) and devices (e.g. audio recorders) used for data collection; if / how the instruments(s) changed over the course of the study	4
Units of study	#12	Number and relevant characteristics of participants, documents, or events included in the study; level of participation (could be reported in results)	5
Data processing	#13	Methods for processing data prior to and during analysis, including transcription, data entry, data management and security, verification of data integrity, data coding, and anonymisation / deidentification of excerpts	4
Data analysis	#14	Process by which inferences, themes, etc. were identified and developed, including the researchers involved in data analysis; usually references a specific paradigm or approach; rationale	5
Techniques to enhance trustworthiness	#15	Techniques to enhance trustworthiness and credibility of data analysis (e.g. member checking, audit trail, triangulation); rationale	5
**Results/findings**			
Syntheses and interpretation	#16	Main findings (e.g. interpretations, inferences, and themes); might include development of a theory or model, or integration with prior research or theory	5-14

For peer review only

Links to empirical data	#17	Evidence (e.g. quotes, field notes, text excerpts, photographs) to substantiate analytic findings	5-14
**Discussion**			
Intergration with prior work, implications, transferability and contribution(s) to the field	#18	Short summary of main findings; explanation of how findings and conclusions connect to, support, elaborate on, or challenge conclusions of earlier scholarship; discussion of scope of application / generalizability; identification of unique contributions(s) to scholarship in a discipline or field	14
Limitations	#19	Trustworthiness and limitations of findings	15
**Other**			
Conflicts of interest	#20	Potential sources of influence of perceived influence on study conduct and conclusions; how these were managed	16
Funding	#21	Sources of funding and other support; role of funders in data collection, interpretation and reporting	16

The SRQR checklist is distributed with permission of Wolters Kluwer © 2014 by the Association of American Medical Colleges. This checklist was completed on 07. June 2019 using https://www.goodreports.org/, a tool made by the EQUATOR Network in collaboration with Penelope.ai

For peer review only

BMJ Open

A qualitative exploration of women's experiences of intramuscular pethidine or remifentanil patient controlled analgesia for labour pain.

Journal:	*BMJ Open*
Manuscript ID	bmjopen-2019-032203.R2
Article Type:	Original research
Date Submitted by the Author:	22-Nov-2019
Complete List of Authors:	Moran, Victoria; UCLAN, ; University of Central Lancashire,<br>Thomson, Gillian; University of Central Lancashire, School of Health<br>Cook, Julie; University of Central Lancashire,<br>Storey, Hannah; University of Central Lancashire,<br>Beeson, Leanne; The University of Birmingham,<br>MacArthur, Christine; University of Birmingham, Public Health<br>Wilson, Matthew; University of Sheffield, Health Services Research
**Primary Subject Heading**:	Obstetrics and gynaecology
Secondary Subject Heading:	Qualitative research, Medical management, Evidence based practice
Keywords:	Maternal medicine < OBSTETRICS, labour, analgesia, pethidine, remifentanil, Pain management < ANAESTHETICS

SCHOLARONE™
Manuscripts

A qualitative exploration of women's experiences of intramuscular pethidine or remifentanil patient controlled analgesia for labour pain

Authors:

Moran Victoria Hall; Thomson Gillian, Cook Julie, Storey Hannah, Beeson Leanne, MacArthur Christine, Wilson Matthew

Moran Victoria Hall College of Health and Wellbeing, University of Central Lancashire, Preston, United Kingdom

Thomson Gillian, Faculty of Health and Wellbeing, University of Central Lancashire, Preston, United Kingdom

Cook Julie, Faculty of Health and Wellbeing, University of Central Lancashire, Preston, United Kingdom

Storey Hannah, Faculty of Health and Wellbeing, University of Central Lancashire, Preston, United Kingdom

Beeson Leanne, Institute of Applied Health Research, University of Birmingham, Birmingham, United Kingdom

MacArthur Christine, Institute of Applied Health Research, University of Birmingham, Birmingham, United Kingdom

Wilson Matthew, School of Health and Related Research, University of Sheffield, Sheffield, United Kingdom

Correspondence to: Dr Victoria Hall Moran
Faculty of Health and Wellbeing, University of Central Lancashire, Preston PR1 2HE
Email: VLMoran@uclan.ac.uk
Telephone: 01772 89 3830

For peer review only

WORD COUNT: 6887

For peer review only - http://bmjopen.bmj.com/site/about/guidelines.xhtml

Objectives

To explore women's experiences of remifentanil or pethidine for labour pain.
and infant feeding behaviours at 6weeks postpartum.

Design

Qualitative postnatal sub-study to the randomised controlled trial of remifentanil intravenous patient controlled analgesia (PCA) versus intramuscular pethidine for pain relief in labour (RESPITE). Semi-structured telephone interviews were conducted at 6 weeks postpartum and thematic analysis was undertaken.

Setting

Women recruited to the RESPITE trial from seven UK hospitals.

Participants

Eighty women consented and 49 (30 remifentanil group and 19 pethidine group) completed the interview.

Results

Eight themes emerged, which encompassed women's antenatal plans for pain management (*Birth Expectations*), through to their future preferences for pain relief (*Reflections for Future Choices*). Many women who used remifentanil felt it provided effective pain relief (*Effectiveness of Pain Relief*), whereas women in the pethidine group expressed more mixed views. Both groups described side-effects, with women using pethidine frequently reporting nausea (*Negative Physiological Responses*) and women using remifentanil describing more cognitive effects (*Cognitive Effects*). Some women who used remifentanil complained of restricted movements due to technical aspects of drug administration, and fear of analgesia running out (*Issues with Drug Administration*). Women described how remifentanil enabled them to maintain their ability to stay focussed during the birth (*Enabling a Sense of Control*). There was little difference in reported breastfeeding initiation and continuation between pethidine and remifentanil groups (*Impact on Infant Behaviour and Breastfeeding*).

Conclusions

Qualitative insights from a follow-up study to a trial which explored experiences of intravenous remifentanil PCA with intra-muscular pethidine injection found that remifentanil appeared to provide effective pain relief whilst allowing women to remain alert and focussed during labour, although as with pethidine, some side-effects were noted. Overall there was little difference in reported breastfeeding initiation and duration between the two groups.

For peer review only

Trial registration: The RESPITE trial is registered at ISRCTN, number ISRCTN29654603.

Keywords: labour, childbirth, analgesia, pethidine, remifentanil, epidural analgesia, pain relief, postpartum, breastfeeding

Contributor statement:

Strengths and limitations of this study

- To our knowledge, this is the first study to explore in depth the experiences and infant feeding behaviours of women using intravenous remifentanil PCA for pain relief in labour
- Substantial number of women (49) interviewed from multiple sites across England
- Fewer women recruited who had used pethidine (19) compared to remifentanil PCA (30)
- A high number of women also received Entonox® prior to randomisation and in combination with pethidine (41; 84%), and some progressed to epidural analgesia (11; 22%), which may have influenced their experiences.

INTRODUCTION

Women's experiences of and response to pain during labour are complex, affected by multiple physiological and psychosocial factors, and consequently many women in labour require some form of pain relief [1]. In line with the rise in the use of technology and intervention in childbirth, pharmacological pain management is widely offered to women in labour on maternity/obstetric units. Traditional pharmacological approaches for pain management include opioid analgesics, epidural analgesia and inhaled analgesia (Entonox®): 50:50 oxygen and nitrous oxide). Epidural analgesia has been shown to be the most effective form of pain relief for labour but may give rise to unwanted side-effects. Systematic reviews of trials have identified that epidural analgesics increase the duration of second stage labour and the risk of instrumental vaginal delivery[2].

It has been reported that up to 25% of women in the UK use pethidine or a similar opioid during labour [3]. Whilst pethidine is known to alleviate labour pain for some women, when compared to placebo no clear differences have been observed in maternal satisfaction with pain relief, or the number of women requesting an epidural analgesic[4]. Pethidine is known to cause maternal side-effects, including nausea, vomiting and sedation[1]. Opioids readily cross the placenta, and neonatal respiratory depression and hypothermia remain concerns[1]. Intrapartum pethidine administration may have a detrimental effect on neonatal behaviour, reducing infant alertness, supressing the rooting and sucking reflex, and may shorten breastfeeding duration[5].

There is increasing interest in the role of remifentanil as an alternative opioid analgesia in labour[6]. Remifentanil is a potent opioid medication with a short half-life, administered by patient-controlled analgesia (PCA), allowing the woman to have the drug as and when it is

For peer review only - http://bmjopen.bmj.com/site/about/guidelines.xhtml

needed, on demand. A systematic review conducted in 2012, based on a few low-quality small trials comparing remifentanil to pethidine administered by various routes, reported a reduction in progression to epidural analgesia with remifentanil[7]. A recent Cochrane review, again including only small poor-quality trials, compared PCA remifentanil to alternative parenteral methods of pain relief in labour, and found that remifentanil (PCA) provided more effective pain relief, and women were more satisfied with pain relief compared to other opioids administered intravenously, intramuscularly or using PCA[8]. Known side-effects of remifentanil include respiratory depression, nausea, pruritus, and decreased heart rate and blood pressure, although there are currently too few studies of remifentanil PCA use in labour to draw definite conclusion with respect to side-effects for women and newborns[8.] Route of analgesia administration has been found to positively impact on women's experiences of labour, with women who self-administered analgesia (intranasal fentanyl) reporting increased autonomy and satisfaction compared to women who relied on the midwife to administer analgesia, who were more often focused on the physical effect of the drug[9]. The influence of remifentanil on infant feeding behaviours remains largely unexplored.

In this paper we report on a qualitative follow-up sub-study to an open-label randomised controlled trial undertaken in 14 UK maternity units. The main aim of the trial (RESPITE) was to compare intravenous remifentanil PCA with intra-muscular pethidine injection to determine whether remifentanil PCA reduced progression to epidural analgesia; also to consider whether it resulted in any adverse maternal or neonatal sequelae. The trial found that remifentanil intravenous PCA halved epidural analgesia conversion rate, compared to intramuscular pethidine[10]. The importance of combining qualitative evidence that explores the views and experiences of service users alongside quantitative evidence to inform safety, effectiveness and cost of interventions to inform antenatal and intrapartum guidelines has been recognised by the World Health Organisation [11,12]. The focus of this follow-up sub-study was to explore women's birth experiences, perceptions of their pain relief, and infant feeding behaviours up to six weeks postpartum, in women allocated to the pethidine or remifentanil trial arms for labour pain.

METHODOLOGY

Design

Full details of the RESPITE trial are reported elsewhere[10]. This sub-study follow-up phase was an adjunct to the trial, and comprised a qualitative exploratory study using semi-structured telephone interviews.

Ethics

Ethics/governance approval was obtained from a National Research Ethics Service Committee (NRES Committee East Midlands - Nottingham 2, reference number: 13/EM/0239), the lead authors' University (STEMH 304), and via the participating Trusts' R&D departments. Participants gave written informed consent before taking part.

Recruitment

Fourteen UK obstetric-led maternity units took part in the RESPITE trial, where all women booked for delivery at participating centres were informed about the study at their antenatal visits. Women were eligible for the trial if they met the following inclusion criteria: 16 years or older and beyond 37 weeks' gestation, with a singleton live baby, in cephalic presentation, who were in established labour (defined as regular painful contractions irrespective of

For peer review only - http://bmjopen.bmj.com/site/about/guidelines.xhtml

cervical dilatation), intending vaginal birth, and were not participating in any other drug trial. For the qualitative sub-study, women who participated in the trial were approached by a research midwife/nurse after childbirth, whilst an in-patient, and provided with a separate information leaflet. Women who were willing to take part in a telephone interview up to approximately 6 weeks post-partum were asked to sign a consent form. Participant contact details for consenting women were securely transferred to the research team, with all identifiable data stored on encrypted university files.

Verbal consent was reconfirmed at the time of interview. Women were recruited over a 16-month period (between June 2015 and September 2016), and maternity units that recruited the most women within a one month period received a £100 Love to Shop voucher.

Interviews
Semi-structured audio-recorded telephone interviews were conducted between 5-8 weeks post-partum by two researchers (JC, HS). Telephone interviews provided participants with the opportunity to disclose intimate and sensitive experiences without feeling uncomfortable. The interviews lasted between 20-30 minutes and explored women's pre-birth expectations for pain relief, their experiences and perceived impact of pain-relief on maternal or infant behaviours, and infant feeding. Questions such as, "How did the pain relief affect your labour? (e.g. emotionally, physically)?", "How did you feel after the birth?", and "How was your baby following the birth?", were posed. All women who took part in an interview received a £10 Love to Shop voucher to thank them for their participation.

Analysis
All interviews were transcribed verbatim, anonymised, and uploaded into NVIVO qualitative software[13]. Data collection and analysis was undertaken concurrently, and Braun and Clark's thematic approach[14] was undertaken to identify patterned meaning across the dataset. Two authors (JC, HS) undertook this work (under the guidance of VHM and GT) through an iterative process of reading, identifying key codes, grouping codes into sub-themes and finally creating themes that were reflective of all views expressed. We paid particular attention to how women's experiences of pethidine and remifentanil, within the context of the trial, compared or differed. Analytical decisions were discussed and shared with all authors both periodically and at the end, to agree on final themes.

Patient and Public Involvement

Members of PRIME (Public and Researchers Involvement in Maternity and Early pregnancy group), a group of maternity service users convened by the University of Birmingham, were involved in reviewing the participant information during study set-up and were represented on the Trial Steering Committee for the RESPITE trial.

RESULTS

Eighty trial participants from seven hospitals agreed to take part in the RESPITE postnatal sub-study. Whilst all 14 sites participating in the RESPITE trial were eligible for the sub-

For peer review only - http://bmjopen.bmj.com/site/about/guidelines.xhtml

study, only seven hospitals successfully consented participants who progressed to interview. Four maternity units received £100 in shopping vouchers as reward for recruiting the most women within a one-month period. Despite numerous contact attempts, 23 women were not interviewed due to women being unavailable during the timeframe of the study, or due to language barriers. Eight women asked to be withdrawn from the study prior to interview. Forty-nine participants (30 remifentanil and 19 pethidine) took part in a telephone interview and were included for analysis.

Demographic information of participants is provided in Table 1. Eight women received pethidine before they entered the trial (trial eligibility stipulated no systemic opioid pain relief within the previous four hours), four of whom were subsequently assigned to the pethidine trial arm, and four to the remifentanil arm. The majority of women (41/49) received gas and air (Entonox®) pain relief in addition to the trial drug. Overall, 11 women progressed to epidural analgesia: 7 from the pethidine arm (conversion rate of 64%), and 4 from the remifentanil arm (conversion rate of 36%), somewhat higher than the conversion rates seen in the main trial (41% and 19% for pethidine and remifentanil respectively)[10]. Of the 11 women who had an epidural analgesic, 5 women had a caesarean section; 2 from the pethidine arm and 3 from the remifentanil arm. A further 3 women had a caesarean section without having had epidural analgesia.

Table 1 Demographic information of study participants

	Remifentanil (n=30)	Pethidine (n=19)	Total (n=49)
**Age of women (years)**			
Mean (SD)	29.3 (5.2)	25.9 (4.5)	28.0 (5.2)
Range	19-39	17-34	17-39
**Ethnicity**			
White	23	14	37
Asian (Indian)	2	1	3
Asian (Pakistani)	2	4	6
Mixed	1	0	1
Other	2	0	2
**Marital status**			
Married/living with partner	30	17	47
Single	0	2	2
**Parity**			
Nulliparous	21	13	34
Parous	9	6	15
**Type of birth**			
Vaginal	26	15	41
Caesarean	4	4	8
**Additional pain relief administered:**			
Epidural analgesia	4	7	11

For peer review only - http://bmjopen.bmj.com/site/about/guidelines.xhtml

Pethidine	4	4	8
Entonox	24	17	41
**Infant feeding intention:**			
Breastfeeding	21	12	33
Formula feeding	6	6	12
No pre-birth plans	3	1	4
**Infant feeding initiation within the first hour post-birth:**			
Breastfeeding	14	8	22
Formula feeding	16	11	27
**Infant feeding status at ~6 weeks**			
Exclusive breastfeeding	7	0	7
Formula feeding	16	14	30
Mixed feeding	7	5	12

In the following section we report on eight inter-linking themes, relating to women's expectations and experiences of pain relief, and the impact of analgesia on women's experiences of infant feeding across the cohort; with similarities and differences in the experiences of different forms of pain relief highlighted. As no stark differences were found between the responses of nulliparous and parous women, no differentiation was made in the findings. A selection of participant quotes are included, together with an identifier that reports that participant number, and either an R (remifentanil) or P (pethidine) suffix to indicate their group allocation.

The eight themes identified encompass women's plans for pain management in the antenatal period (*Birth Expectations*), through to how their experiences of using either remifentanil or pethidine may influence their future preferences for pain relief in labour (*Reflections for Future Choices*). Four themes centred around women's physiological and cognitive responses to the analgesics (*Negative Physiological Response*; *Cognitive Effects*), and reflections on how effective pethidine and remifentanil were for pain relief (*Effectiveness of Pain Relief*) or pain management (*Enabling a Sense of Control*). Additional themes highlighted common issues with the administration of the analgesics which may have impacted negatively on women's experiences (*Issues with Drug Administration*), and their reflections on whether they felt the analgesics had an influence on their newborns and the establishment of breastfeeding (*Impact on Infant Behaviour and Breastfeeding*).

Birth Expectations

There were wide variations in women's approaches to birth planning. These ranged from some women having no clear plans; *'there wasn't any plans, no*' (14R) or how women '*just went*

For peer review only - http://bmjopen.bmj.com/site/about/guidelines.xhtml

For peer review only

with the flow' (36R), to having very specific intentions; '*Yes I did a lot of planning, I knew exactly what I wanted*' (50R). Often women's approaches to labour were related to their previous childbirth experiences. Prior experiences of unfulfilled birth plans often led women to adopt a more flexible perspective due to the uncertainty of childbirth:

> Last time I was determined I wasn't going to have an epidural and then things didn't quite go according to plan so I had an epidural, so this time I decided to be, you know to go more with an open mind, because things can't be planned as much as you would like. (36R)

The lack of a specific birth plan did not mean that women held no preferences or opinions regarding pain relief. Many women had actively researched pain relief options in a variety of ways, such as through antenatal classes, internet searches, and discussions with friends and family, although only two women had any prior knowledge of remifentanil. Some women considered pethidine to be the best available option:

> Well I'd looked at all the different types of pain relief beforehand and decided it was the one that I thought would be the best, so the pethidine. (15P)

However, others were less inclined to use pethidine due to the potential impact on the baby:

> It was really just the fact that it [pethidine] crossed over [to] the baby, and like I didn't really want him coming out sleepy. (55P)

Almost all the women interviewed were initially opposed to having an epidural analgesic due to reasons such as needle phobia, prior negative experience of epidural analgesia and vicarious reports of risk:

> I'd heard so many stories of people having nerve damage and complications. (16P)

For a few of the women, their reticence reflected cultural expectations of how childbirth should be managed:

> I think it's a culture thing. Like, originally my parents are from [country], they're very against it [epidural]. I don't know if it's a myth or if there's any, or there is any research to back it up but they say it can cause lots of problems in the future and stuff like that. So we've kind of just been brought up around that. I've always kind of been told, 'Oh yeah, you know don't get that'. (33P)

Negative physiological responses

Women from both trial arms reported what they considered to be side-effects and negative impacts of the analgesia on their physiological responses. While pethidine has a well-known sedative effect, for some of the women in our study this was not related to effective pain relief, and rather became problematic when it rendered them incapable of staying awake in labour:

> Well I just remember them giving me an injection and […] it just made me, literally just trying not to fall asleep, that's how it felt. Like my eyes were really heavy and I were just nodding off but I was still in so much pain it just made things a bit worse if I'm honest because it didn't feel like it was numbing the pain at all it just literally made me want to go to sleep and I couldn't. [...] … (73P – progressed to epidural analgesia)

For peer review only - http://bmjopen.bmj.com/site/about/guidelines.xhtml

Some women across both trial arms experienced drowsiness to the point that it interfered with their autonomy and mobility during labour:

> I was lying down and they told me not to move too much because obviously because it makes you that drowsy and that sleepy that it's best to stay in one position and just to press a button when you feel the pain. (45R)

Many of the women in the trial already had their mobility restricted by monitors, oxytocin infusion etc, and some of these women found that the delivery of remifentanil through a PCA device requiring continuous intravenous access further restricted their mobility:

> I found it [labour] very painful and I couldn't walk around, I couldn't really be on all fours very easily. All the things that I had imagined, I just couldn't do because I was attached to the machine as well so yeah [...] (65R)

Several women in the pethidine arm reported experiencing vomiting or nausea, compared to only one woman in the remifentanil group. However, this woman reflected that it was difficult to disentangle the effects of remifentanil from the side-effects of other analgesia:

> Perhaps a little bit drowsy because I know I was a bit in and out, and I was quite sick but I don't know if that was gas and air more than the actual drug. (74R)

Furthermore, for some women, their adverse physiological responses to remifentanil were only experienced when the analgesia *'ran out'*:

> The only thing I would say is because I was on it for quite a long time, the drip actually ran out and when the drip ran out, I had quite bad sickness and like body-shaking, my whole body was shaking, also it kind of, I think it got blocked at one point as well so that happened twice where like I was violently throwing up everywhere. (60R)

On some occasions, as reflected in the following quote, symptoms associated with remifentanil led some women to cease using this form of pain relief:

> ...the first couple of times I did it okay, from the first go I didn't, I said I don't feel right and then after that I couldn't focus enough to even press the button when I needed to... I was just not with it, and then my hubby said, 'Oh can I press it for you?', but the midwife said, 'Oh no it's got to be…', it had to be me that did it. But I just couldn't, so I think in total I only pressed it probably four or five times and then decided to stop because I just didn't, I just didn't like it at all. (36R)

Effectiveness of pain relief

There were mixed views regarding the effectiveness of pethidine for pain relief. Some women were happy with the pain relief obtained from pethidine; '*it was brilliant. I literally didn't feel a thing until after she was born* (38P)'. Women were satisfied with its effectiveness, particularly when used in combination with other pain-relief methods, e.g. gas and air:

> When I just had the gas and air it just felt like, wasn't really working at all, wasn't really doing anything but then obviously the two together just sort of took the edge off a little

For peer review only - http://bmjopen.bmj.com/site/about/guidelines.xhtml

> bit. Obviously I remember still feeling a bit of pain but it's sort of bearable if you like, a bit more bearable. Yeah, definitely felt the difference. (52P)

From a counter-perspective, half of the women who received pethidine reported that their pain relief had been ineffective. These women complained about how they could *'feel everything'* and how it had made *'no difference'* to the intensity of their contractions:

> I didn't find the pethidine any help. It didn't help me at all [...] like, she said that it would like ease the contractions off, you know like the pain, but I didn't feel, I could still feel the pain when she, after the pethidine got injected in I could still feel everything [...] It might help other women but it didn't help me. (79P)

In contrast, only a few women found remifentanil ineffective as pain relief. However, this minority of accounts were generally related to delivery of the medication, or its inability to prevent pain caused by the baby's position in utero:

> As the labour progressed, because my son was back-to-back, a lot of the pain was in my back and I found it helped with the contractions but not necessarily the back pain in between the contractions. (58R)

Overall, there was a notable enthusiasm for remifentanil among participants. Some women described experiencing 'no pain' after its administration, whereas others referred to how it made the pain more bearable:

> Yes, it did. It definitely did help because before that it was just getting really unbearable. It was just so unbearable, I just couldn't, I felt as if I wanted to die [laughs], I know that's an exaggeration but that's how it feels at that point where you just can't bear that pain but when I took that medication or pain relief, it took that pain away. Obviously as soon as I felt that contraction coming, press the buzzer and it's like more than half of it were gone and it were just, it just slowly eases it all off and it were quite good. It was bearable then. (59R)

Enabling a sense of control

One of the key benefits of remifentanil highlighted by women was how it provided pain relief *and* maintained their ability to stay alert, awake or focussed during the birth:

> I still remember being very alert and having to listen to what the midwives were saying, you know push and everything, so I was still very alert and able to do what I needed to do [...] (44R)

For some women, being connected to a cannula did not substantially restrict their ability to move from the bed during labour, which, as reflected in the quote below, could have a positive impact on women's agency and self-efficacy:

> That's what I wanted to do anyway. I wanted a water birth. I wanted to be active. As soon as I got into labour anyway, before I had that medication, I didn't want to be on the bed, I hated being on the bed, being stuck with, restricted. I didn't like it, I just wanted to be able to move and it started giving me a little bit more confidence as well, you know, I just, not even, obviously I didn't care, I just wanted to get her out. I couldn't have done it without that [remifentanil], I really couldn't. (50R)

For peer review only - http://bmjopen.bmj.com/site/about/guidelines.xhtml

Women often needed time and practice to get into the rhythm of delivering remifentanil via the PCA. Once mastered, several women talked about how the PCA enabled them to retain a sense of control by actively managing their own labour:

> But it was just so nice that you could be in control of it and it was one of those things that I didn't have to have it all the time. So, for example, with contractions, I'd press the button on the first, you know, with one contraction, and then I wouldn't have it for a couple of times because I'd be in control of my body, if that makes sense. [...] And for me, it was just what I wanted, I could feel, I was in control of me, I was in control of my body. I knew what was going on in my surroundings. (1R)

This capacity for self-regulation was supported by the immediate impact of remifentanil on women's physiological responses:

> But I noticed as well, when I pressed the buzzer, it worked straightaway, you know, the pain… [...] I just noticed like when I pressed the buzzer, you know, when I pressed the buzzer, you know, for pain relief, it was like instant, yes, the pain just like went. (12R)

The ability to maintain focus was strongly linked to the short-acting effect of remifentanil. The fact that each dose of remifentanil wore off quickly was experienced as empowering. One participant found that discontinuing use of the PCA quickly re-established the control she felt she was lacking:

> I really feel like when it came to the time of needing to push and things, when you're having to do sort of certain things at certain times, I needed to, I would have struggled if I had still been taking it because I couldn't kind of concentrate on anything, whereas because I'd stopped it and it was pretty much out of my system I was able to focus more. So I don't think it was in my system for very long. (36R)

On occasion, these positive effects were contrasted with women's previous experience of using pethidine:

> It was, obviously, because you've got control of it. So, you know, you were able to press it when you needed it. Whereas like, if you have the pethidine and that, once you've been given it you've got no control over how it's going to work. (14R)

While some of the women who had received pethidine made positive comments about its impact on their sense of control during labour, this was often in the context of providing respite to manage their labour pains:

> So I had the pain relief for a good few hours which had kind of given me a bit of respite actually because I was getting quite tired and quite, I really needed pain relief so when I got that then it just gave me a little bit of respite...(6R)

Cognitive effects

Some women, particularly in the remifentanil group, reported cognitive effects consistent with systemic opioids. For some, this was a negative experience, associated with a sense of

For peer review only - http://bmjopen.bmj.com/site/about/guidelines.xhtml

vulnerability, where they felt unable to focus or process external stimuli and could, as reflected below, negate the potential salutary effects of medication use on labour pains:

> I was, I don't really know the technical term, almost knocked out by it, it made me really sort of, I don't know if that's the right word but I was really out of it, I couldn't focus, it was almost […] like it was too strong for me and they reduced the dose of it but […] the midwife was trying to ask me questions and give me instructions as they do, it's ongoing, and I really couldn't […] focus because I was almost sort of not in the room as it were, so for me that sort of, sort of negated any positive effect it would have had on the pain relief side of things. (36R)

Whereas while other women described feeling *'out of it'*, or *'away with the fairies'* after remifentanil administration, this was not always seen as being entirely negative:

> I would say it did take away my control and my sort of presence in the process so, you know at one point I could hear my mum and husband talking about me but couldn't voice an answer to them...Now that did mean that I was, you know, in a lovely place, and I wasn't feeling any pain at that point but it meant that I was almost removed from the labour process. (58R)

A number of women in the remifentanil group also reflected on how their confused or altered state of mind was observed by their birth partners:

> I mean my husband said that I was talking about the car insurance, I was saying to him, 'Is the car insured?'...I was having a conversation about the car insurance and he was like, this is a weird time to talk about the car insurance (56R).

> I was in, kind of in a completely different zone I think. My mum and husband were there and said that I'd gone from being in incredible pain to just really blissed out. (58R)

For both pain relief medications, women likened the effects of the analgesia to heavy alcohol consumption:

> To be honest I felt incredibly drunk or to the same effect. (55P)

> It was like being very, very, drunk. (76R)

A further negative impact reported by women with respect to both forms of pain relief related to how the analgesia had impacted on their memories of labour and childbirth:

> I must admit within, literally within, I've no idea because I was out of it. [...] So then, again I don't know how long I was on it for. […] Or maybe she was crying. I can't remember. I can't remember, sadly. (44R)

Issues with drug administration
On some occasions, difficulties were experienced due to the potential or actual unavailability of remifentanil. For example, some women described feeling *'stressed out'* due to concerns about whether the analgesia (drip bag) would run out:

For peer review only - http://bmjopen.bmj.com/site/about/guidelines.xhtml

> The pain relief it was brilliant, really, really good for about three hours and then the effects started to wear off. I don't know if the contractions were getting worse but I was clicking it a lot more than I had originally and I was running out of it and I was getting a bit stressed out because I was worried that I was going to run out. (32R)

In some instances, remifentanil was only reinstated after what women felt was a long delay (if at all), leaving them unexpectedly without pain relief:

> …but to leave someone without, there probably was a bit of pain relief in me but that ten minutes was a long ten minutes. Yeah, it's quite traumatising. (63R)

One woman reported that the issue of remifentanil running out had contributed to her using epidural analgesia:

> I ended up going on to have an epidural because my medication had finished out and what happened was my baby's heart rate kept dipping in and out. So they were a bit concerned and, obviously, I was still in pain after that, then I wasn't fully dilated. So I needed something to, to take the edge off, basically, so I did go in for an epidural, yes. (7R)

Several women also reported difficulties in opportunities to receive pain relief when the cannula became detached:

> I'd managed to rip my thingy out, the cannula thing so I didn't have it through the last bit basically because she said, 'Oh I can't get it put back in, you'll just have to go on gas and air for the last bit'. (44R)

For some women the physiological effects of the remifentanil interfered with their ability to use the PCA to deliver the pain relief for each contraction:

> The other thing was, when I was using the button this is, it goes straight to your head, you know, and I was so woozy, when my next contraction would come, I would forget to press the button. And that contraction would feel absolutely dire because obviously the contraction before it had taken the edge off, and you know the midwife had to keep telling me [name] you need to press the button, and I was like, oh OK, OK. But I was in and out of it if I'm honest, but it was good, it was good. (48R)

Whereas for others, as reflected in the quote below, the stability of the cannula directly affected their mobility:

> When I lifted up my hand it never went in properly, it had come up with an error message. So I had to make sure my hand was down when I pressed the button, which is a bit difficult when you're in contractions...No, I told them it wasn't working but they just told me to keep my hand down. (10R)

Reflections for future choices

Some of the women who received pethidine reported that they would opt for this form of pain relief in a future birth, even where the analgesia had not been wholly effective:

For peer review only - http://bmjopen.bmj.com/site/about/guidelines.xhtml

> It didn't get rid of the pain, but I would have it again...Yes, I wouldn't even think about it, I'd just have it next time...Obviously I'd go for the gas and air again if I could and then if it was like I was told I couldn't have it then I would go for the pethidine. (69P)

In converse, others were adamant that they would not use pethidine again, i.e. *'I hope I never have to have it* [pethidine] *again'* (55P), and would where possible choose an alternative such as remifentanil:

> If hell froze over and I got pregnant again, I'd definitely have, I'd request remifentanil from the get go. (25P)

Similar mixed responses were also evident amongst women who received remifentanil. A number of women referred to how they would *'definitely'* use it again:

> I definitely felt I needed it so I would definitely have it again and I would definitely choose the same form of pain relief if it was offered to me again. I felt like it really worked for me. It definitely gave me what I needed and what I wanted from pain relief so I would definitely do the same again. (39R)

And would recommend it to others:

> I've also said to any other friends...if you are offered it [remifentanil], go for it because it's brilliant. (1R)

Whereas other women, notably all of whom had experienced adverse responses, would either request pethidine or make concerted efforts to not use pain relief:

> Gas and air, yeah I'd have gas and air again, unless I needed it epidural no. I don't think any of the trial drugs, or the drug I had before I wouldn't have. So just gas and air...But me, I don't think I'd do it again. It didn't agree with me, and yeah I don't think I'd take it myself. (29R)

Impact on infant behaviour and breastfeeding

The majority of women (33/49) expressed a prior intention to breastfeed their babies, whilst 12 women intended to formula feed and four had made no plans prior to their baby's birth (see Table 1). Within the first hour of birth 47% (14/30) of women in the remifentanil group and 42% (8/19) of women in the pethidine group breastfed. By the time of interview (approximately 6 weeks postnatal), 47% (14/30) of women in the remifentanil group and 26% (5/19) in the pethidine group were either exclusively breastfeeding or mixed feeding their infants. A number of reasons were given for an earlier than expected cessation of breastfeeding, including the level of support received, feeding difficulties such as infant tongue-tie and inverted nipples, and health problems for the mother (e.g. lupus, heart condition, mental health issues) or the baby (need for antibiotics, cleft palate, inadequate weight gain). None of the participants felt that the pain relief medication they received influenced their decision to cease breastfeeding.

Women in both groups reported feeling exhausted immediately following the birth, and how this affected their initial interactions with their baby. However, as the effects of analgesia, and

For peer review only - http://bmjopen.bmj.com/site/about/guidelines.xhtml

particularly remifentanil, dissipate after a short period of time, these complaints are likely to be attributed to the length and exertions associated with labour, rather than pain relief analgesia per se:

> The first day, obviously, the night that he was born, I didn't [have skin to skin], after he was born I was just asleep. When my mum come that evening, that afternoon, so she was there and my husband was there, and that day I was asleep. (11P)

> Uncomfortable, tired, drowsy like even the nurses had to take over sometimes to feed my son in the night because I was that drowsy still and I couldn't, I just couldn't take any control, it was a case of I was falling asleep with him on me and stuff like that because I was just so tired. (17R)

Other women felt so weak that they needed help from their birth partners to hold their babies:

> So he was born, they were saying, give him milk, they put him on to my breast and I was giving him the milk but my arms didn't have that much strength to hold him, my husband had to help me out. (11P)

One woman who had progressed to epidural analgesia reflected on her increasing frustration at not being able to breastfeed her baby:

> I was so exhausted. I couldn't really move because of my epidural and I was getting really tired and a bit emotional and everything so it wasn't, breastfeeding wasn't working basically. (44R)

DISCUSSION

This study aimed to explore women's experiences of remifentanil and pethidine use for pain relief during labour and infant feeding behaviours up to approximately 6 weeks postpartum. The findings revealed that whilst many women had actively researched pain relief options prior to their birth and had either formulated birth plans or had decided to adopt a more flexible approach to pain management, most were unfamiliar with the opioid remifentanil until they were informed about the trial. The majority of women who had used remifentanil felt that it had provided effective pain relief. This was not the case with women in the pethidine group, who had more mixed views, with many reporting that pethidine was completely ineffective at relieving their pain. This was consistent with the findings of the RESPITE trial, which showed that women's views of their pain relief effectiveness and average pain scores were significantly better in the remifentanil arm[10]. These insights also support a recent study by Fleet and colleagues[9], who compared women's use of pethidine and fentanyl (delivered on an intranasal or subcutaneous basis). Fleet's study found that women who received fentanyl provided more positive, and less negative responses when compared to those who received pethidine[9]. However, it is important to note that while effective pain management has become an essential component of the care plan for expectant mothers, women's perceptions of pain are affected by physiological and/or psychological issues (e.g. fear, anxiety)[15], as well as the nature of the woman-provider relationship[16].

For peer review only - http://bmjopen.bmj.com/site/about/guidelines.xhtml

Women using either form of pain relief reported experiencing a number of side-effects, although these tended to differ according to the analgesia used. Women using pethidine frequently reported feeling nauseated, consistent with the findings of Fleet and colleagues[9], whereas this was only reported by one woman who had used remifentanil. Women in both groups reported that the opioid made them feel drowsy, although this seemed to be less pronounced in the remifentanil group. These insights support the RESPITE trial findings[10] and the work of others such as Volikas and Male[17] in regard to the sedative effects of opioid-based forms of pain relief.

A number of negative comments arose relating to the nature of remifentanil administration. Women in the remifentanil group described how the PCA restricted their movements, and for a small number this resulted in pain (as a result of technically flawed intravenous cannulation) and frustration. Others experienced anxiety or difficulties due to the potential or actual unavailability of remifentanil. Women reported feeling anxious about whether the analgesia (drip bag) would run out, and on occasions when it did, they experienced significant delay in its reinstatement. On some occasions women considered that this may have contributed to their progression to epidural analgesia. Other qualitative research has also highlighted how opioids administered too late, or which wore off too early, created fear and anxiety[9,18]. It is important to note that these technical difficulties are not inevitable components of PCA, and are amenable to resolution.

One of the key advantages of remifentanil highlighted by women was how it provided pain relief and maintained their ability to stay focussed during the birth. Several women referred to how PCA enabled them to use pain relief selectively, as required. In this sense, they saw remifentanil as an opportunity to stay in control and actively manage their own labour (not just the pain), helping them to find reference points for their own understanding of their progression and tolerance to pain. The ability to maintain focus was probably related to the short-acting effect of remifentanil, which enhanced their sense of control. The association between the use of pharmacological pain relief and increased sense of control has also been reported by others[9,12,19,20].

A further notable finding, as reflected by others[9,21] relates to women experiencing a cognitive impact from the analgesics. Some women reported feeling disconnected from their labour and unable to focus or process external stimuli. Similar findings were reported in a study by Whitburn and colleagues to explore women's experiences of labour pain. They found that an inability to retain focus during labour was associated with self-judgement and negative perspectives of pain[22]. Some women felt that remifentanil impacted on their memory and affected their ability to recall their baby's birth. While this was not found in Fleet and colleagues' study[9], these differences may be related to additional factors such as levels of fear, and dosage of the medication, or the fact that more women in our study received remifentanil than pethidine.

In relation to infant feeding, the main trial found no difference in breastfeeding within an hour of birth in the two trial arms[10]; with a similar pattern noted amongst those in this sub-study. Forty-seven percent of women in the remifentanil and 42% of women in the pethidine arms initiated breastfeeding within the first hour after birth. At around 6 weeks postnatal, the rates of 'any' breastfeeding were 26% for women who used pethidine compared to 46% of women in the remifentanil group. While these variations may be attributable to the level of postnatal support for breastfeeding across the different areas, the findings do support those by Fleet and colleagues[23] in that women who used remifentanil had fewer breastfeeding difficulties. These

For peer review only - http://bmjopen.bmj.com/site/about/guidelines.xhtml

differences may relate to the differential effects of analgesia on infant behaviours, which may impact on establishment of sustained breastfeeding[24]. However, as there are many biopsychosocial factors that can influence the mother's infant feeding decisions, further research is needed.

To our knowledge, this is the first study to explore the experiences of women using intravenous remifentanil PCA for pain relief in labour in depth, by formal, qualitative methodology. The study comprised a qualitative sub-study to a multi-centre RCT that compared intravenous remifentanil PCA with intra-muscular pethidine for pain relief in labour. The strengths of this study are that to date, there is minimal qualitative research to understand women's experiences of opioid use during labour, and the substantial number of interviews conducted in multiple sites across England. The limitations relate to the relatively fewer women recruited who were randomised to pethidine, and it may be that women who held stronger views towards the use of analgesia (positive or negative) took part in the study. The high attrition rates for the study highlight challenges intrinsic to early recruitment into postpartum studies. For example women may not have been fully cognisant of what they were agreeing to, and difficulties are encountered in organising data collection when participants are dealing with the demands of a new baby. It is also important to reflect that 41 (84%) of our sample received Entonox® prior to randomisation and in combination with pethidine, and 11 (22%) progressed to epidural analgesia, which may have influenced their experiences.

CONCLUSIONS

Qualitative insights from a follow-up sub-study to an open-label trial that compared intravenous remifentanil PCA with intra-muscular pethidine injection highlighted valuable differences. Overall, more women found remifentanil to be an effective pain relief, when compared with the mixed views of women who received pethidine. All women experienced side-effects although these differed between groups, with a number of the difficulties associated with remifentanil related to drug administration rather than impact of the analgesia. While negative experiences of remifentanil included restricted movements due to drug administration, a key advantage of this form of pain relief was how its short-lasting nature enabled women to remain focussed during the birth. Overall, while there was little difference in breastfeeding initiation rates, there was an indication that women who received remifentanil were more likely to continue breastfeeding. While these variations may be attributable to different numbers of women included from the trial groups and the level of postnatal support for breastfeeding across the different geographical areas, it may be that women who used remifentanil had fewer breastfeeding difficulties.

List of abbreviations

PCA (Patient Controlled Analgesia), RESPITE (A Randomised Controlled Trial of Remifentanil intravenous patient Controlled analgesia (PCA) versus intramuscular pethidine for pain relief in labour), ISRCTN (International Standard Randomised Controlled Trial Number), R&D (Research and Development).

For peer review only - http://bmjopen.bmj.com/site/about/guidelines.xhtml

DECLARATIONS

Funding statement

This work was supported by the Healthy Children Project, Inc. The RESPITE trial was funded by the National Institute for Health (NIHR) Research Clinician Scientist Award (NIHR ref: CS-11-030). CM is part funded by NIHR Collaborations for Leadership in Applied Health Research and Care West Midlands. The views expressed are those of the author(s) and not necessarily those of the NIHR or the Department of Health and Social Care.

Data sharing statement

The data (deidentified quotes) are available from the corresponding author upon reasonable request.

Competing interests

The authors declare no competing interests.

Contributor statement

The postnatal sub-study was conceived by VHM and GT and they secured funding to conduct it. VHM and GT performed the literature search and were responsible for the study design, with input from MW, CM and LB. Study conduct and data collection was led by VHM, GT, JC and HS with contributions from MW, CM and LB. Study analysis and was performed by JC and HS with supervision from VHM and GT. All authors were involved in data interpretation. Writing of the paper was led by VHM, GT, JC and HS with input from MW, CM and LB.

Acknowledgements

Professor Jane Daniels contributed to the trial design and conduct.

We thank all the women who participated in the RESPITE postnatal sub-study and thank Dr Karin Cadwell and Kajsa Brimdyr of the Healthy Children Project for their support in funding his study. Finally, we express our gratitude to our National Health Service colleagues who supported recruitment for the study.

References

1 Jones L, Othman M, Dowswell T, Alfirevic Z, Gates S, Newburn M, Jordan S, Lavender T, Neilson JP. *Pain management for women in labour: an overview of systematic reviews. Cochrane Database of Systematic Reviews 2012, Issue 3. Art. No.: CD009234. DOI: 10.1002/14651858.CD009234.pub2.*

2 Anim-Somuah M, Smyth RMD, Cyna AM, Cuthbert A. Epidural versus non-epidural or no analgesia for pain management in labour. Cochrane Database of Systematic Reviews

For peer review only - http://bmjopen.bmj.com/site/about/guidelines.xhtml

For peer review only

2018, Issue 5. Art. No.: CD000331. DOI: 10.1002/14651858.CD000331.pub4.

3 Redshaw M, Henderson J. Safely Delivered: a National Survey of Women's Experience of Maternity Care 2014. Oxford: The National Perinatal Epidemiology Unit, University of Oxford, 2015.
4 Smith LA, Burns E, Cuthbert A. Parenteral opioids for maternal pain management in labour. Cochrane Database of Systematic Reviews 2018, Issue 6. Art. No.: CD007396. DOI: 10.1002/14651858.CD007396.pub3
5 Burchell, T., Coster, S. and Norman, I., 2016. The effect of intrapartum pethidine on breastfeeding: a scoping review. *Evidence Based Midwifery*, *14*, pp.49-56.
6 Kranke, P., Girard, T., Lavand'homme, P., Melber, A., Jokinen, J., Muellenbach, R.M., Wirbelauer, J. and Hönig, A., 2013. Must we press on until a young mother dies? Remifentanil patient controlled analgesia in labour may not be suited as a "poor man's epidural". *BMC pregnancy and childbirth*, *13*(1), p.139.
7 Schnabel A, Hahn N, Broscheit J, Muellenbach RM, Rieger L, Roewer N, et al. Remifentanil for labour analgesia: a meta-analysis of randomised controlled trials. European Journal of Anaesthesiology (EJA). 2012;29(4):177-85.
8 Weibel, S., Jelting, Y., Afshari, A., Pace, N.L., Eberhart, L.H., Jokinen, J., Artmann, T. and Kranke, P., 2017. Patient-controlled analgesia with remifentanil versus alternative parenteral methods for pain management in labour. *Cochrane Database of Systematic Reviews*, (4).
9 Fleet J, Jones M, Belin I: Taking the alternative route: Women's experience of intranasal fentanyl, subcutaneous fentanyl or intramuscular pethidine for labour analgesia. Midwifery 2017, 53:15-19.
10 Wilson, M.J., MacArthur, C., Hewitt, C.A., Handley, K., Gao, F., Beeson, L., Daniels, J. and Group, R.T.C., 2018. Intravenous remifentanil patient-controlled analgesia versus intramuscular pethidine for pain relief in labour (RESPITE): an open-label, multicentre, randomised controlled trial. *The Lancet*, *392*(10148), pp.662-672
11 Downe, S., Finlayson, K., Tunçalp, Ö. and Metin Gülmezoglu, A., 2016. What matters to women: a systematic scoping review to identify the processes and outcomes of antenatal care provision that are important to healthy pregnant women. *BJOG: An International Journal of Obstetrics & Gynaecology*, *123*(4), pp.529-539.

12 Thomson, G., Feeley, C., Moran, V.H., Downe, S. and Oladapo, O.T., 2019. Women's experiences of pharmacological and non-pharmacological pain relief methods for labour and childbirth: a qualitative systematic review. *Reproductive health*, *16*(1), p.71

13 NVIVO Software https://www.qsrinternational.com/nvivo

14 Braun, V. and Clarke, V., 2006. Using thematic analysis in psychology. *Qualitative research in psychology*, *3*(2), pp.77-101.
15 Lundgren I, Dahlberg K: Women's experience of pain during childbirth. Midwifery 1998, 14(2):105-110.
16 Karlsdottir S, Halldorsdottir S, Lundgren I: The third paradigm in labour pain preparation and management: the childbearing woman's paradigm. Scandinavian Journal of Caring Sciences 2014, 28(2):315-327.
17 Volikas I, Male D. A comparison of pethidine and remifentanil patient-controlled analgesia in labour. Int J Obstet Anesth. 2001;10(2):86-90.
18 Nikkola E, Läärä A, Hinkka S, Ekblad U, Kero P, Salonen M: Patient-controlled epidural analgesia in labor does not always improve maternal satisfaction. Acta Obstet Gynecol Scand 2006, 85(2):188-194

For peer review only - http://bmjopen.bmj.com/site/about/guidelines.xhtml

19 Doering K, Patterson J, Griffiths C: Japanese women's experiences of pharmacological pain relief in New Zealand. Women and Birth 2014, 27(2):121-125
20 Lally, J.E., Thomson, R.G., MacPhail, S. and Exley, C., 2014. Pain relief in labour: a qualitative study to determine how to support women to make decisions about pain relief in labour. *BMC pregnancy and childbirth*, *14*(1), p.6.
21 Jantjes L, Strumpher J, Kotze W: The experience of childbirth in first-time mothers who received narcotic analgesics during the first stage of labour. Curationis 2007, 30(2):82-90.
22 Whitburn LY, Jones LE, Davey M-A, Small R. Women's experiences of labour pain and the role of the mind: an exploratory study. Midwifery. 2014;30(9):1029-35
23 Fleet, J., Jones, M., Belan, I. The influence of intrapartum opioid use on breastfeeding experience at 6 weeks post partum: a secondary analysis. Midwifery 2017 50, 106–109
24 Ransjö-Arvidson, A.B., Matthiesen, A.S., Lilja, G., Nissen, E., Widström, A.M. and Uvnäs-Moberg, K., 2001. Maternal analgesia during labor disturbs newborn behavior: effects on breastfeeding, temperature, and crying. *Birth*, *28*(1), pp.5-12.

For peer review only

For peer review only - http://bmjopen.bmj.com/site/about/guidelines.xhtml

Reporting checklist for qualitative study.

Based on the SRQR guidelines.

Instructions to authors

Complete this checklist by entering the page numbers from your manuscript where readers will find each of the items listed below.

Your article may not currently address all the items on the checklist. Please modify your text to include the missing information. If you are certain that an item does not apply, please write "n/a" and provide a short explanation.

Upload your completed checklist as an extra file when you submit to a journal.

In your methods section, say that you used the SRQRreporting guidelines, and cite them as:

O'Brien BC, Harris IB, Beckman TJ, Reed DA, Cook DA. Standards for reporting qualitative research: a synthesis of recommendations. Acad Med. 2014;89(9):1245-1251.

		Reporting Item	Page Number
**Title**			
	#1	Concise description of the nature and topic of the study identifying the study as qualitative or indicating the approach (e.g. ethnography, grounded theory) or data collection methods (e.g. interview, focus group) is recommended	1

For peer review only

**Abstract**			
	#2	Summary of the key elements of the study using the abstract format of the intended publication; typically includes background, purpose, methods, results and conclusions	2
**Introduction**			
Problem formulation	#3	Description and signifcance of the problem / phenomenon studied: review of relevant theory and empirical work; problem statement	3-4
Purpose or research question	#4	Purpose of the study and specific objectives or questions	4
**Methods**			
Qualitative approach and research paradigm	#5	Qualitative approach (e.g. ethnography, grounded theory, case study, phenomenolgy, narrative research) and guiding theory if appropriate; identifying the research paradigm (e.g. postpositivist, constructivist / interpretivist) is also recommended; rationale. The rationale should briefly discuss the justification for choosing that theory, approach, method or technique rather than other options available; the assumptions and limitations implicit in those choices and how those choices influence study conclusions and transferability.	4

For peer review only

		As appropriate the rationale for several items might be discussed together.	
Researcher characteristics and reflexivity	#6	Researchers' characteristics that may influence the research, including personal attributes, qualifications / experience, relationship with participants, assumptions and / or presuppositions; potential or actual interaction between researchers' characteristics and the research questions, approach, methods, results and / or transferability	4
Context	#7	Setting / site and salient contextual factors; rationale	4
Sampling strategy	#8	How and why research participants, documents, or events were selected; criteria for deciding when no further sampling was necessary (e.g. sampling saturation); rationale	4
Ethical issues pertaining to human subjects	#9	Documentation of approval by an appropriate ethics review board and participant consent, or explanation for lack thereof; other confidentiality and data security issues	4
Data collection methods	#10	Types of data collected; details of data collection procedures including (as appropriate) start and stop dates of data collection and analysis, iterative process, triangulation of sources / methods, and modification of procedures in response to evolving study findings; rationale	4

For peer review only

Data collection instruments and technologies	#11	Description of instruments (e.g. interview guides, questionnaires) and devices (e.g. audio recorders) used for data collection; if / how the instruments(s) changed over the course of the study	4
Units of study	#12	Number and relevant characteristics of participants, documents, or events included in the study; level of participation (could be reported in results)	5
Data processing	#13	Methods for processing data prior to and during analysis, including transcription, data entry, data management and security, verification of data integrity, data coding, and anonymisation / deidentification of excerpts	4
Data analysis	#14	Process by which inferences, themes, etc. were identified and developed, including the researchers involved in data analysis; usually references a specific paradigm or approach; rationale	5
Techniques to enhance trustworthiness	#15	Techniques to enhance trustworthiness and credibility of data analysis (e.g. member checking, audit trail, triangulation); rationale	5

Results/findings

Syntheses and interpretation	#16	Main findings (e.g. interpretations, inferences, and themes); might include development of a theory or model, or integration with prior research or theory	5-14

For peer review only

Links to empirical data	#17	Evidence (e.g. quotes, field notes, text excerpts, photographs) to substantiate analytic findings	5-14
**Discussion**			
Intergration with prior work, implications, transferability and contribution(s) to the field	#18	Short summary of main findings; explanation of how findings and conclusions connect to, support, elaborate on, or challenge conclusions of earlier scholarship; discussion of scope of application / generalizability; identification of unique contributions(s) to scholarship in a discipline or field	14
Limitations	#19	Trustworthiness and limitations of findings	15
**Other**			
Conflicts of interest	#20	Potential sources of influence of perceived influence on study conduct and conclusions; how these were managed	16
Funding	#21	Sources of funding and other support; role of funders in data collection, interpretation and reporting	16

The SRQR checklist is distributed with permission of Wolters Kluwer © 2014 by the Association of American Medical Colleges. This checklist was completed on 07. June 2019 using https://www.goodreports.org/, a tool made by the EQUATOR Network in collaboration with Penelope.ai

For peer review only
